# *Schistosoma mansoni* infection alters the host pre-vaccination environment resulting in blunted Hepatitis B vaccination immune responses

**DOI:** 10.1371/journal.pntd.0011089

**Published:** 2023-07-05

**Authors:** Roshell Muir, Talibah Metcalf, Slim Fourati, Yannic Bartsch, Jacqueline Kyosiimire-Lugemwa, Glenda Canderan, Galit Alter, Enoch Muyanja, Brenda Okech, Teddy Namatovu, Irene Namara, Annemarie Namuniina, Ali Ssetaala, Juliet Mpendo, Annet Nanvubya, Paul Kato Kitandwe, Bernard S. Bagaya, Noah Kiwanuka, Jacent Nassuna, Victoria Menya Biribawa, Alison M. Elliott, Claudia J. de Dood, William Senyonga, Priscilla Balungi, Pontiano Kaleebu, Yunia Mayanja, Matthew Odongo, Jennifer Connors, Pat Fast, Matt A. Price, Paul L. A. M. Corstjens, Govert J. van Dam, Anatoli Kamali, Rafick Pierre Sekaly, Elias K. Haddad

**Affiliations:** 1 Division of Infectious Diseases and HIV Medicine, Department of Medicine, Drexel University College of Medicine, Philadelphia, Pennsylvania, United States of America; 2 PATRU, School of Medicine, Emory University, Atlanta, Georgia, United States of America; 3 Ragon Institute of MGH, MIT, and Harvard, Cambridge, Massachusetts, United States of America; 4 MRC/UVRI and LSHTM Uganda Research Unit, Entebbe, Uganda; 5 Department of Medicine, Allergy and Immunology, University of Virginia, Charlottesville, Virginia, United States of America; 6 UVRI-IAVI HIV Vaccine Program, Entebbe, Uganda; 7 Department of Immunology and Molecular Biology, School of Biomedical Sciences, Makerere University, College of Health Sciences, Kampala, Uganda; 8 Department of Epidemiology and Biostatistics, School of Public Health, Makerere University, College of Health Sciences, Kampala, Uganda; 9 Department of Clinical Research, London School of Hygiene and Tropical Medicine, London, United Kingdom; 10 Department of Cell and Chemical Biology, Leiden University Medical Center, Leiden, Netherlands; 11 International AIDS Vaccine Initiative, New York, New York, United States of America; 12 Pediatric Infectious Diseases, Stanford University School of Medicine, Palo Alto, California, United States of America; 13 Department of Epidemiology and Biostatistics, University of California at San Francisco, San Francisco, California, United States of America; 14 Department of Parasitology, Leiden University Medical Center, Leiden, the Netherlands; 15 IAVI, New York, New York, United States of America, and Nairobi, Kenya; University of Liverpool, UNITED KINGDOM

## Abstract

Schistosomiasis is a disease caused by parasitic flatworms of the *Schistosoma spp*., and is increasingly recognized to alter the immune system, and the potential to respond to vaccines. The impact of endemic infections on protective immunity is critical to inform vaccination strategies globally. We assessed the influence of *Schistosoma mansoni* worm burden on multiple host vaccine-related immune parameters in a Ugandan fishing cohort (n = 75) given three doses of a Hepatitis B (HepB) vaccine at baseline and multiple timepoints post-vaccination. We observed distinct differences in immune responses in instances of higher worm burden, compared to low worm burden or non-infected. Concentrations of pre-vaccination serum schistosome-specific circulating anodic antigen (CAA), linked to worm burden, showed a significant bimodal distribution associated with HepB titers, which was lower in individuals with higher CAA values at month 7 post-vaccination (M7). Comparative chemokine/cytokine responses revealed significant upregulation of CCL19, CXCL9 and CCL17 known to be involved in T cell activation and recruitment, in higher CAA individuals, and CCL17 correlated negatively with HepB titers at month 12 post-vaccination. We show that HepB-specific CD4^+^ T cell memory responses correlated positively with HepB titers at M7. We further established that those participants with high CAA had significantly lower frequencies of circulating T follicular helper (cTfh) subpopulations pre- and post-vaccination, but higher regulatory T cells (Tregs) post-vaccination, suggesting changes in the immune microenvironment in high CAA could favor Treg recruitment and activation. Additionally, we found that changes in the levels of innate-related cytokines/chemokines CXCL10, IL-1β, and CCL26, involved in driving T helper responses, were associated with increasing CAA concentration. This study provides further insight on pre-vaccination host responses to *Schistosoma* worm burden which will support our understanding of vaccine responses altered by pathogenic host immune mechanisms and memory function and explain abrogated vaccine responses in communities with endemic infections.

## Author summary

Schistosomiasis drives host immune responses for optimal pathogen survival, potentially altering host responses to vaccine-related antigen. Chronic schistosomiasis and co-infection with hepatotropic viruses are common in countries where schistosomiasis is endemic. We explored the impact of *Schistosoma mansoni* (*S*. *mansoni*) worm burden on Hepatitis B (HepB) vaccination of individuals from a fishing community in Uganda. We demonstrate that higher schistosome-specific antigen (circulating anodic antigen, CAA) concentration pre-vaccination, is associated with lower HepB antibody titers post-vaccination at month 7. We show higher pre-vaccination levels of CCL17 in instances of high CAA that negatively associate with HepB antibody titers month 12 post-vaccination and coincided with lower frequencies of circulating T follicular helper cell populations (cTfh), proliferating antibody secreting cells (ASCs), and higher frequencies of regulatory T cells (Tregs). We also show that monocyte function is important in HepB vaccine responses, and high CAA is associated with alterations in the early innate cytokine/chemokine microenvironment. Our findings suggest that in individuals with high CAA and likely high worm burden, schistosomiasis can create an environment that is polarized against optimal host immune responses to the vaccine, which puts many endemic communities at risk for infection against HepB and other diseases that are preventable by vaccines.

## Introduction

While vaccines have reshaped public health and saved millions of lives from the threat of many infectious diseases [1], inadequate immune responses to vaccination remain a challenge, with a range of contributary causes, such as chronic illnesses, including those stemming from helminth worm infections [2–4]. Schistosomiasis is a neglected tropical disease (NTD) caused by parasitic flatworms from three main *Schistosoma* spp. that often develops into a chronic infection in endemic populations. An estimated four million people in Uganda are infected with *S*. *mansoni*, about 55% of the population are at risk of infection [5,6] and treatment with Praziquantel (PZQ) is the only safe and effective protocol for treatment. *Schistosoma* has been suggested to increase the virulence of hepatotropic viruses [7] and exacerbate resultant hepatic pathology [8]. Hepatitis B (HepB) is a life-threatening disease cause by HBV and is highly endemic in Uganda. The prevalence is approximately 4.3% with an increased incidence in the Lake Victoria fishing communities, where the risk of *Schistosoma* infection and sexually transmitted diseases such as HepB is high [9,10]. In 2022, the Ugandan ministry of health incorporated early childhood vaccination programs against HepB into its existing program [11] but despite these interventions, 52% of Ugandan adults are still infected with HBV [12]. While vaccination against HepB can provide efficient protective immunity [13], 5–10% of individuals do not mount an effective immune response to HepB vaccination, including those with chronic diseases [14]. There is a need for improved understanding of the immunological responses to infections such as *Schistosoma* during vaccination, to establish the extent of protective responses in compromised host immune systems.

*Schistosoma* spp. induce strongly skewed host T helper 2 (Th2) responses during infection and go on to induce regulatory T cell (Treg) responses which are important in controlling T helper-induced inflammation [15,16]. Specifically, during the chronic phase, continual exposure to the soluble egg antigen (SEA) of schistosome eggs in the host tissues drives immune-pathology and a pro-Th2 cytokine microenvironment [17,18]. Persistence of these skewed Th2 responses could potentially inhibit important T follicular helper (Tfh) responses, which are critical for B cell help during vaccination [19,20], and polarize cytokine environments that cause alterations in vaccine-induced antibody responses. While less is known about Tfh responses in *Schistosoma*, Tfh cells in the periphery are elevated in *S*. *japonicum*-infected individuals compared to healthy controls, and in chronic, but not acute infection, correlates with IL-21 and SEA-specific B cell responses [21,22]. Further understanding of how schistosomiasis alters the Tfh host response during vaccination holds promise for new strategies to ensure protective vaccine responses.

While there have been studies on *Schistosoma* infection and HepB vaccination responses [23,24], there is less known about the impact of *S*. *mansoni* worm burden on multiple vaccine-related immune parameters, and the underlying actions of immune mediators affecting these responses during HepB vaccination, including biomarkers of ineffective vaccine responses. Knowledge of these mechanisms could instruct future vaccination programs as against viruses such as HIV and SARS-CoV-2 [25,26]. While the standard for diagnosis of *Schistosoma* is the examination of stool and/or urine for eggs [27], symptoms of schistosomiasis are not caused only by the body’s reaction to the eggs, but also by the worms themselves. To improve understanding of immunological responses during vaccination and *Schistosoma* infection, we evaluated samples from a cohort of fisherfolk participants that were enrolled in a simulated vaccine efficacy trial (SiVET) in Uganda, where the ENGERIX-B HepB vaccine, a known and trusted vaccine against HepB [28] was administered to HepB antigen-negative individuals. The individuals from this community were regularly exposed to *Schistosoma* and would potentially have varying levels of worm burden.

After the conclusion of the clinical study, we carried out this sub study to characterize soluble and cellular immune factors at pre- and post-immunization timepoints in PZQ-treated individuals that had high and low serum worm burden which we determined using a circulating anodic antigen (CAA) assay [29–31] and also explored associations which could indicate how vaccine responses can be altered in individuals with a high worm burden. The CAA assay is a quantitative indication of worm antigen regurgitated into the bloodstream of the host, therefore CAA levels in the serum not only indicates there was an ongoing infection at the time of sampling, but also implies the presence of living worms and hence a direct association with worm burden [32,33]. We demonstrated that *S*. *mansoni* infection is associated with significantly lower HepB vaccine-specific humoral responses and showed these responses coincided with alterations in cytokines and chemokines that are important for T helper and Treg cell recruitment and function. We propose that a high *S*. *mansoni* worm burden pre-vaccination, could reshape the baseline immune environment, and modulate the response to Hep B vaccination.

## Results

### Pre-vaccination *S*. *mansoni* infection negatively impacts Hepatitis B antibody titers post-vaccination

**Fig 1:**
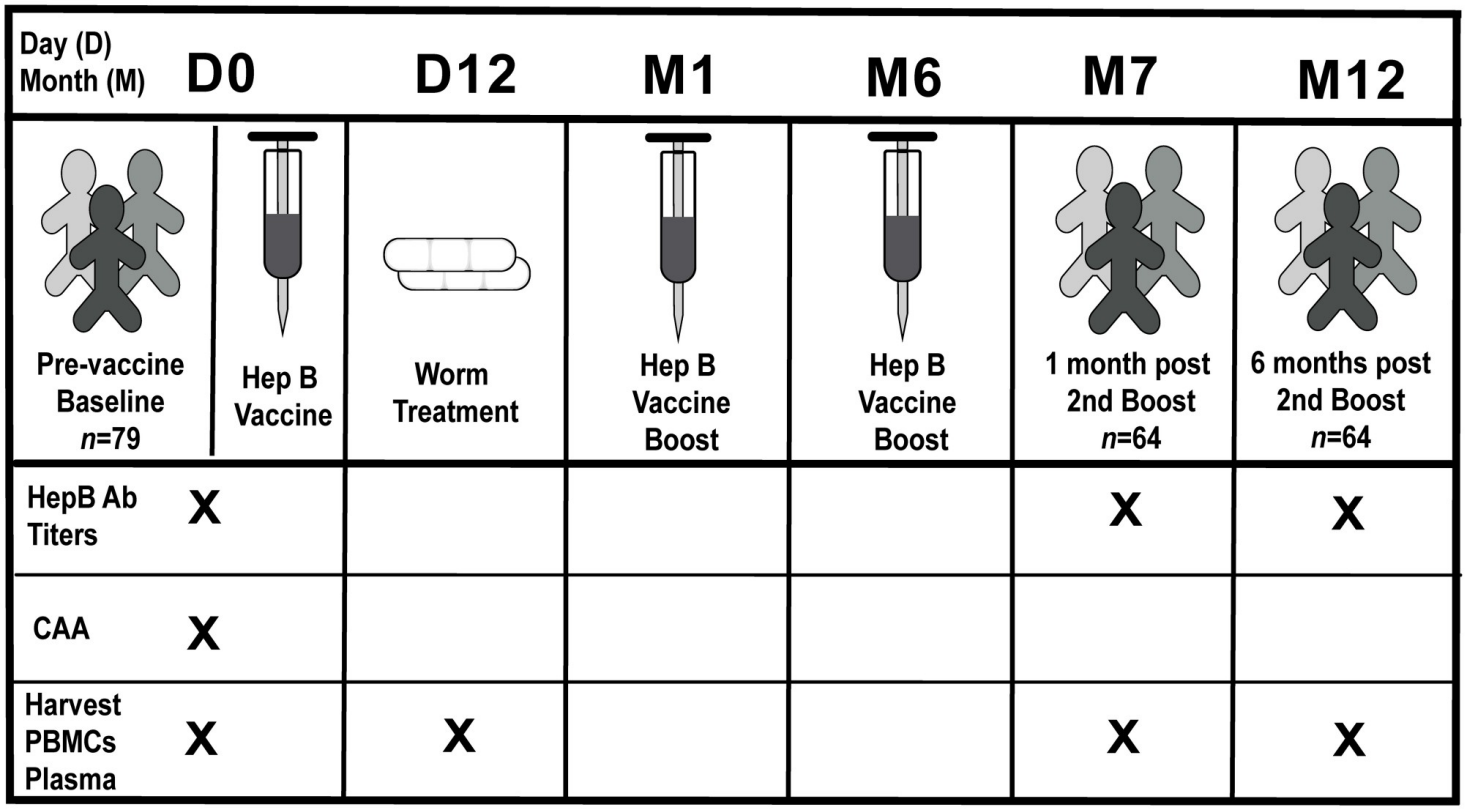
Clinical study design. Participants enrolled in the clinical study (n = 79, four donor samples were unavailable for analysis in this study) were vaccinated at baseline [(pre-vaccination) day 0 (D0)] and received two boosters at month 1 (M1) and month 6 (M6). Sera samples were collected at D0, month 7 post-vaccination (M7) (one month post-booster 2) and month 12 post-vaccination (M12) (six months post-booster 2), and PMBCs and plasma were collected at D0, D12, M7 and M12 for subsequent analysis. Stool samples were collected at D0, D3 or D7 for egg count analysis. Egg positive individuals were treated at day 12 post-vaccination (D12) with Praziquantel (PZQ). X denotes corresponding samples collected at that timepoint.

Samples were obtained from participants in the simulated vaccine efficacy trial (SiVET), a community-based program conducted by Ugandan research institutes to assess the implementation of efficacy trial procedures to further the understanding of vaccine study design in a cohort of HepB-vaccinated individuals. Participants were recruited in Uganda among fishing communities on the northern shores of Lake Victoria where schistosomiasis is endemic, and individuals that tested egg positive at the start of the trial were treated with Praziquantel (PZQ) on day 12 (D12) (Fig 1 and S1 Table). HepB antigen-negative individuals were enrolled in the study over a 13-month period and various samples were collected at multiple timepoints (Fig 1 and S1 Table). After completion of the trial, we were able to conduct our sub study on specimen (pre- and post-vaccination) for seventy-five participants who received all 3 doses of the HepB vaccine and gave consent to for subsequent analyses.

**Fig 2:**
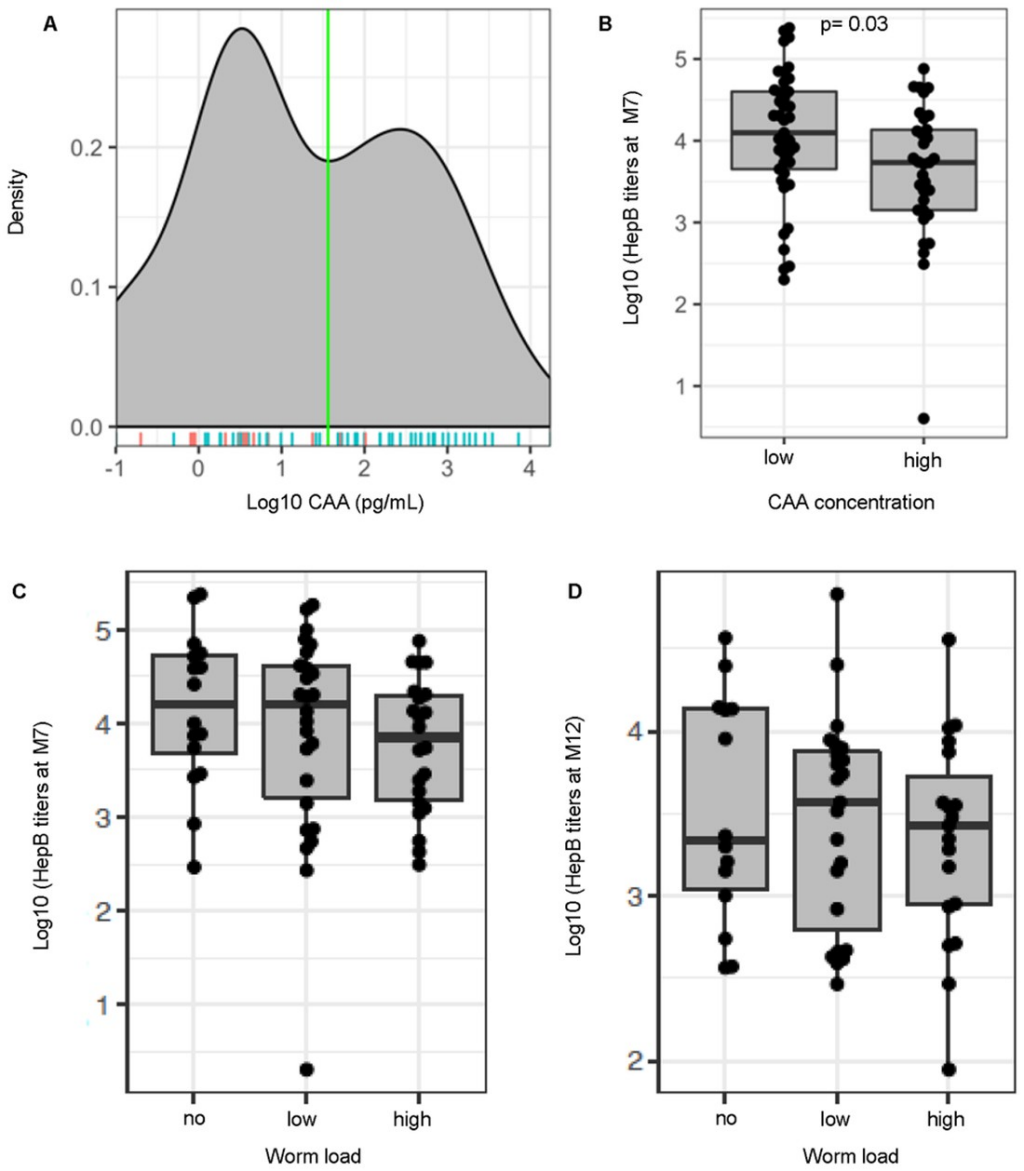
Higher CAA concentration pre-vaccination is associated with a reduction in Hepatitis B vaccine titers. (A) Density of Schistosome-specific antigen values (CAA [pg/ml]) analyzed in serum samples from participants pre-vaccination (D0). A binomial distribution was fitted to the CAA values and a maximum-likelihood was used to identify the optimum cutoff separating the two modes of the CAA values. Two groups of participants were then identified based on CAA values [low CAA (<36pg/mL CAA), n = 41; high CAA (≥36pg/mL CAA), n = 33]. Male (blue line), n = 53; Female (red line), n = 21 (B) Hepatitis B (HepB) titers (log pg/mL) determined by commercial immunoassays for individuals at M7 post-vaccination plotted to compare low and high CAA. Participants were further defined based on CAA concentration values using methodology from the CAA assay that allowed detection of samples as low as 3pg/mL [non-infected [no] (<3pg/mL CAA), low CAA [low] (3–100 pg/mL CAA), and high CAA [high] (>100pg/mL CAA)]. HepB titers for individuals at (C) Month 7 post-vaccination [non-infected n = 16, low CAA, n = 26 and high CAA, n = 22] and (D) Month 12 post-vaccination [non-infected, n = 14, low CAA, n = 23, high CAA, n = 19] plotted to compare CAA. Boxplots show median values (horizontal line), interquartile range (box) and 95% confidence interval (whiskers).

To evaluate quantitative data on worm burden that we could correlate with immunological responses pre- and post- vaccination, we used a circulating anodic antigen (CAA) detection assay [29–31,34] carried out with the serum of enrolled participants collected pre-vaccination (Fig 1 and S1 Table). CAA data indicate an ongoing infection, implying the presence of living *Schistosoma* worms, and there is a direct association of CAA serum level with worm burden [32,33]. Results show that CAA values displayed a bimodal distribution. The cutoff separating the two modes was estimated by maximum-likelihood which revealed that participants could be separated into those with a CAA concentration of <36 pg/mL (non-infected and lower worm burden); and those with a CAA concentration of ≥36 pg/mL (higher worm burden) (Fig 2A). HepB antibody titers (anti-HBs) in the serum at D0, M7 (1 month post-2^nd^ boost) and M12 (6 months post-2^nd^ boost) was also measured to look at levels pre-vaccine, as well as short-term and longer-term post-vaccination antibody responses after completion of the immunization series (Fig 1 and S1 Table). CAA values from these two groups of participants were significantly associated with HepB titers measured at month 7 (M7), where low CAA was associated with a high titer, and high CAA with a low titer (log10 fold change (FC) = -0.43, 95% confidence interval (CI) [-0.053, -0.81]; P *=* 0.028) (Fig 2B). The same trend between HepB titers and the two CAA groups was observed after adjusting for age and sex (log10FC = -0.38, 95% CI [0.059, -0.81]; P = 0.089).

As *Schistosoma* infection modulates host immunity [35–37], individuals were further stratified into three groups using methodology from the CAA assay that allowed detection of samples as low as 3pg/mL to identify non-infected (no) (CAA <3 pg/mL), as well as low CAA concentration (low worm burden) (CAA 3–100 pg/mL) and high CAA concentration (high worm burden) (CAA >100 pg/mL) (S1 Table) [38], in order to compare immunological changes pre- and post- vaccination during *Schistosoma* infection, and importantly with differing levels of worm burden. While no significant associations with HepB titers were observed between these 3 groups at M7 and M12 (Fig 2C and 2D), we were interested in exploring other vaccine-related immune parameters known to be important during vaccination [39,40].

### Plasma cytokines/chemokines involved in lymphocyte migration and activation are significantly higher in *S*. *mansoni* infection pre-vaccination and persist at month 12 post-vaccination

**Fig 3:**
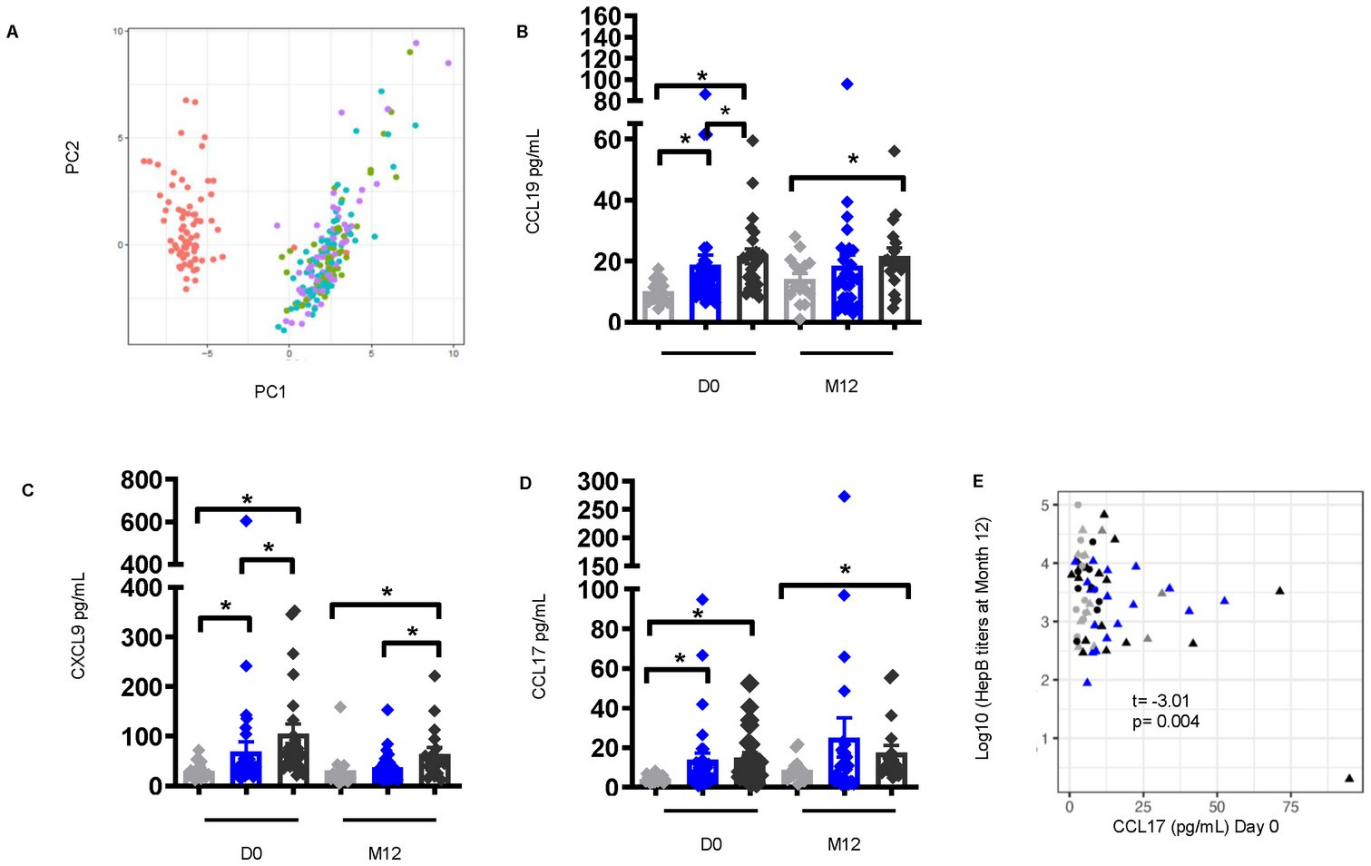
Elevated levels of plasma cytokines/chemokines involved in lymphocyte cell migration and activation pre-vaccination in individuals with *S*. *mansoni* infection persist at month 12 post-vaccination. (A) Principal component analysis of plasma cytokines/chemokines pre-vaccination and after Hepatitis B vaccination was conducted and the first (PC1) and second (PC2) principal components were used to plot samples based on their plasma cytokines/chemokines profiles. Each dot corresponds to a sample and colors denote the timepoint the sample was collected. [D0- red, D3- green, D7- blue, and M12- purple]. Plasma levels of (B) CCL19, (C) CXCL9, and (D) CCL17 [non-infected pre-vaccination (D0), n = 19, low CAA, n = 32, and high CAA, n = 24; M12 post-vaccination, n = 15, low CAA, n = 29, and high CAA, n = 19]. Data shown as ± SEM. * P ≤ 0.05. Wilcoxon rank-sum test performed on non-infected vs. low CAA, or non-infected vs. high CAA, or low CAA vs. high CAA within each time point D0 or M12. Non-infected- light grey, low CAA—blue, and high CAA—dark grey. (E) Scatter plot of Hepatitis B titers at M12 as a function of plasma CCL17 cytokine levels at D0 [non-infected, n = 19, low CAA, n = 32, high CAA, n = 24]. Linear regressions were fit between Hepatitis B titers and the cytokines adjusted for sex and student t-tests were used to evaluate for the significance of the association. t (t-statistic). P ≤ 0.05 was considered significant. (Shape: circle-females, triangle-males; color: grey- non-infected, black-low CAA, blue- high CAA).

The cytokine/chemokine environment dictates the immune response during *Schistosoma* infection [35]. To elucidate any patterns of cytokine/chemokine production between infected groups, plasma samples from non-infected and *S*. *mansoni*-infected individuals were analyzed using a multiplex cytokine/chemokine platform of more than 65 soluble factors. Principal component analysis (PCA) analysis showed that cytokine/chemokine levels pre-vaccination, clustered separately from post-vaccination time points, suggesting an association between HepB vaccination and major changes in circulating cytokines/chemokines that are sustained over time (up to 6 months after the last immunization, Fig 3A). We identified that pre-vaccination systemic levels of CCL19 (no vs. low P = 0.004, no vs. high P<0.0001, low vs. high P = 0.040), CXCL9 (no vs. low P = 0.012, no vs. high P<0.0001, low vs. high P = 0.002), and CCL17 (no vs. low P = 0.002, no vs. high P<0.0001 (Fig 3B–3D) were significantly higher in individuals with low and high CAA compared to non-infected at D0. Of interest, the levels of these cytokines (CCL19, no vs. high P = 0.040; CXCL9, no vs. high P = 0.004, low vs. high P = 0.031; CCL17, no vs. high P = 0.012) remained significantly elevated in individuals with high CAA at M12 post-vaccination (Fig 3B–3D). The relationship between alterations in these cytokines/chemokines and the levels of HepB titers was evaluated to identify potential biomarkers associated with the humoral response to vaccination, and pre-vaccination (t = -3.01, P = 0.004) levels of CCL17 showed a significant negative correlation with HepB titers at M12 (Fig 3E)., These observations imply that a high worm burden in *S*. *mansoni* infection is associated with alterations of the host cytokine/chemokine response to vaccination, and suggests an important role for CCL17 pre-vaccination in modulating the long term HepB vaccine response.

### Frequencies of circulating T follicular helper cell populations are significantly lower pre-vaccination which is sustained post-vaccination in *S*. *mansoni* infection and coincides with higher frequencies of regulatory T cells in individuals with high CAA concentration

**Fig 4:**
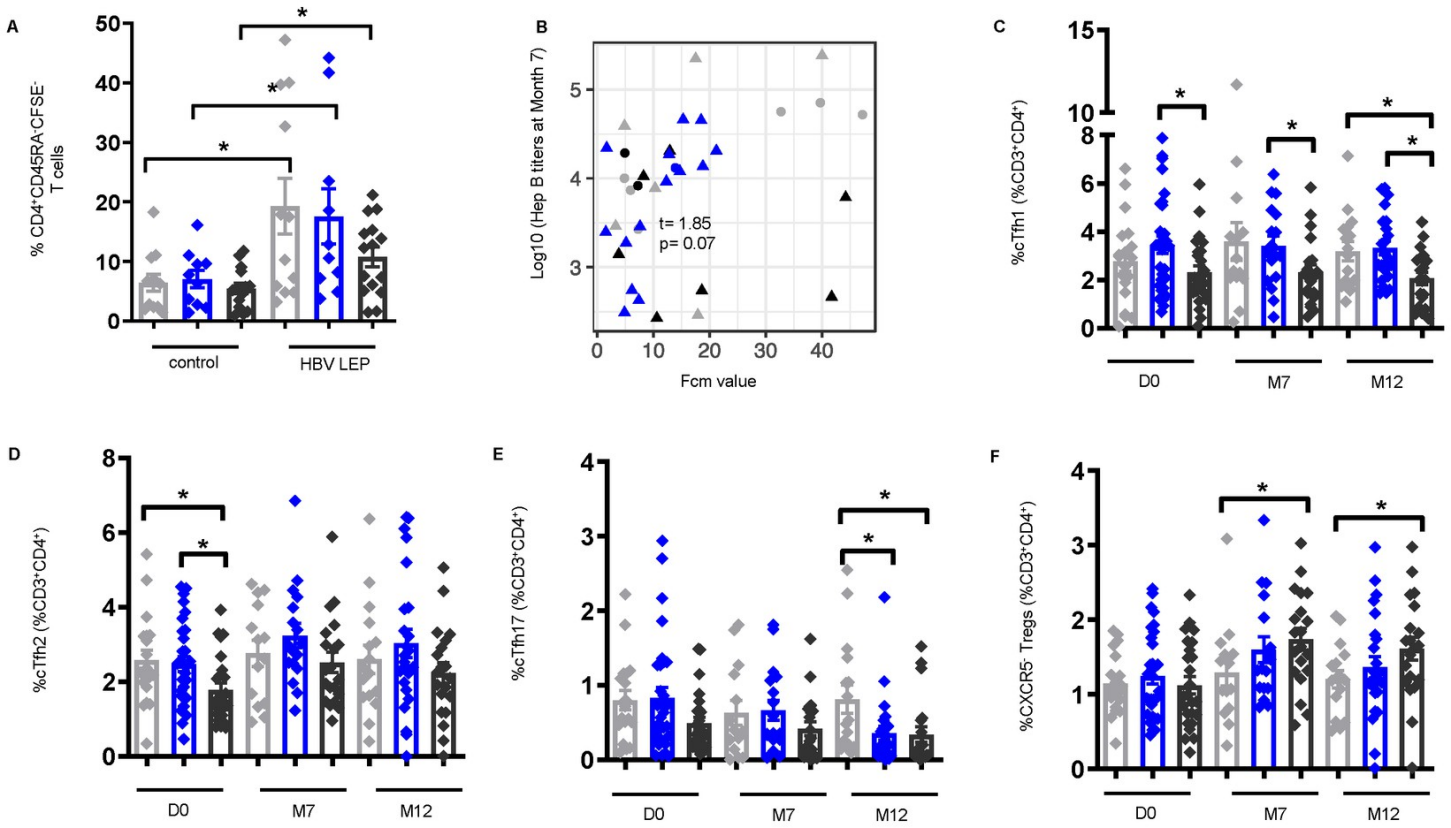
Frequencies of circulating follicular helper (cTfh) cells are lower pre- and post-vaccination in *S*. *mansoni* infection with concurrent higher frequencies of regulatory T cells (Tregs) in individuals with high CAA concentration. (A) Frequencies of CFSE^-^ CD4^+^CD45RA^-^ memory T (CD4^+^ mem) cells identified by flow cytometry of PBMCs at M7 post vaccination [non-infected, n = 12, low CAA, n = 10, and high CAA, n = 15] stimulated for 6 days with hepatitis B long envelope protein peptide (HBV LEP) or DMSO control (control). Non-infected- light grey, low CAA—blue, and high CAA—dark grey. Wilcoxon matched-pairs signed rank test performed on control vs. HBV LEP for each non-infected, or low CAA, or high CAA. Data shown as ± SEM. *P ≤ 0.05. (B) Linear regressions fit between Hepatitis B titers and CFSE^-^ CD4^+^ mem T cells, adjusted for sex, and student t-tests evaluated for the significance of the association. t (t-statistic). P ≤ 0.05 was considered significant. (Shape: triangle-Male, circle-Female; color: grey- non-infected, black- low CAA, blue- high CAA). Frequencies of CD3^+^CD4^+^CD45RA^-^CD25^-^CXCR5^+^ populations: (C) cTfh1 [CXCR3^+^], (D) cTfh2 [CXCR3^-^CCR6^-^], (E) cTfh17 [CXCR3^-^CCR6^+^], and (F) CXCR5^-^Tregs [CD3^+^CD4^+^CD45RA^-^CD127^-^CD25^+^Foxp3^+^CXCR5^-^] identified by flow cytometry of PBMCs pre-vaccination (D0) [non-infected, n = 19, low CAA, n = 31, high CAA, n = 25], M7 post-vaccination [non-infected, n = 14, low CAA, n = 17, high CAA, n = 20], and M12 post-vaccination [non-infected, n = 16, low CAA, n = 24, high CAA, n = 20]. Data shown as ± SEM. *P ≤ 0.05. Wilcoxon rank-sum test performed on non-infected vs. low CAA, or non-infected vs. high CAA, or low CAA vs. high CAA for each time point separately D0, M7, or M12. Non-infected- light grey, low CAA—blue, and high CAA—dark grey.

Cellular immunological changes are key in modulating effective vaccine-specific humoral responses [2]. To determine the impact of *S*. *mansoni* infection on HepB-specific memory T cells, PBMCs from non-infected and *S*. *mansoni*-infected individuals at M7 were treated with a Hepatitis B long envelope protein (HBV LEP) peptide, and CFSE^-^ T cells analyzed by flow cytometry were identified as vaccine-specific. CFSE^-^ memory CD4^+^ T were significantly higher after peptide stimulation compared to control (DMSO); and while not significant, lower frequencies of HBV-LEP-specific CFSE^-^ memory CD4^+^ T cells were observed in high worm burden individuals compared to non-infected (Fig 4A). Interestingly, a positive association was observed between CFSE^-^ memory CD4^+^ T cells and HepB titers (t = 1.85, P = 0.073) (Fig 4B), which highlights the importance of the vaccine-specific CD4^+^ memory T cell response in driving antibody responses to the vaccine.

We further assessed frequencies of circulating T follicular helper (cTfh) populations [41], memory CD4^+^ T cells critically involved in providing B cell help in infection and vaccination [42–44], from the PBMCs of non-infected and *S*. *mansoni*-infected individuals by flow cytometry (S1 Fig). Frequencies of cTfh1 (low vs. high P = 0.020) and cTfh2 cells (no vs. high P = 0.012, low vs. high P = 0.014) pre-vaccination (Fig 4C and 4D), were significantly lower in individuals with high CAA; and cTfh1 cells remained significantly lower at M7 (low vs. high P = 0.031) and M12 (no vs. high P = 0.030, low vs. high P = 0.004) post-vaccination (Fig 4C). While differences between non-infected and *S*. *mansoni*-infected groups for cTfh17 cells pre-vaccination did not reach statistical significance, they were significantly lower in *S*. *mansoni*-infected individuals at M12 post-vaccination (no vs. low P = 0.024, no vs. high P = 0.006) (Fig 4E). Importantly, these alterations were specific to cTfh cells, as no statistically significant changes between groups were observed at any of the time points for non-cTfh cells (S2 Fig). By contrast, frequencies of CXCR5^-^ regulatory T cells (Tregs), that were similar between non-infected and *S*. *mansoni*-infected groups pre-vaccination, were significantly higher at M7 (no vs. high P = 0.039) and M12 (no vs. high P = 0.047) post-vaccination in individuals with high CAA (Fig 4F). These results suggest that frequencies of cTfh cells, which are important for instructing B cells to produce antibodies, are significantly altered in instances of high worm burden, and the concomitant increase in Tregs could support the theory that worm burden may have an impact on the interplay between these two subsets.

### Antibody-secreting B cells are significantly lower in instances of high worm burden and is associated with Hepatitis B antibody titers at month 12

**Fig 5:**
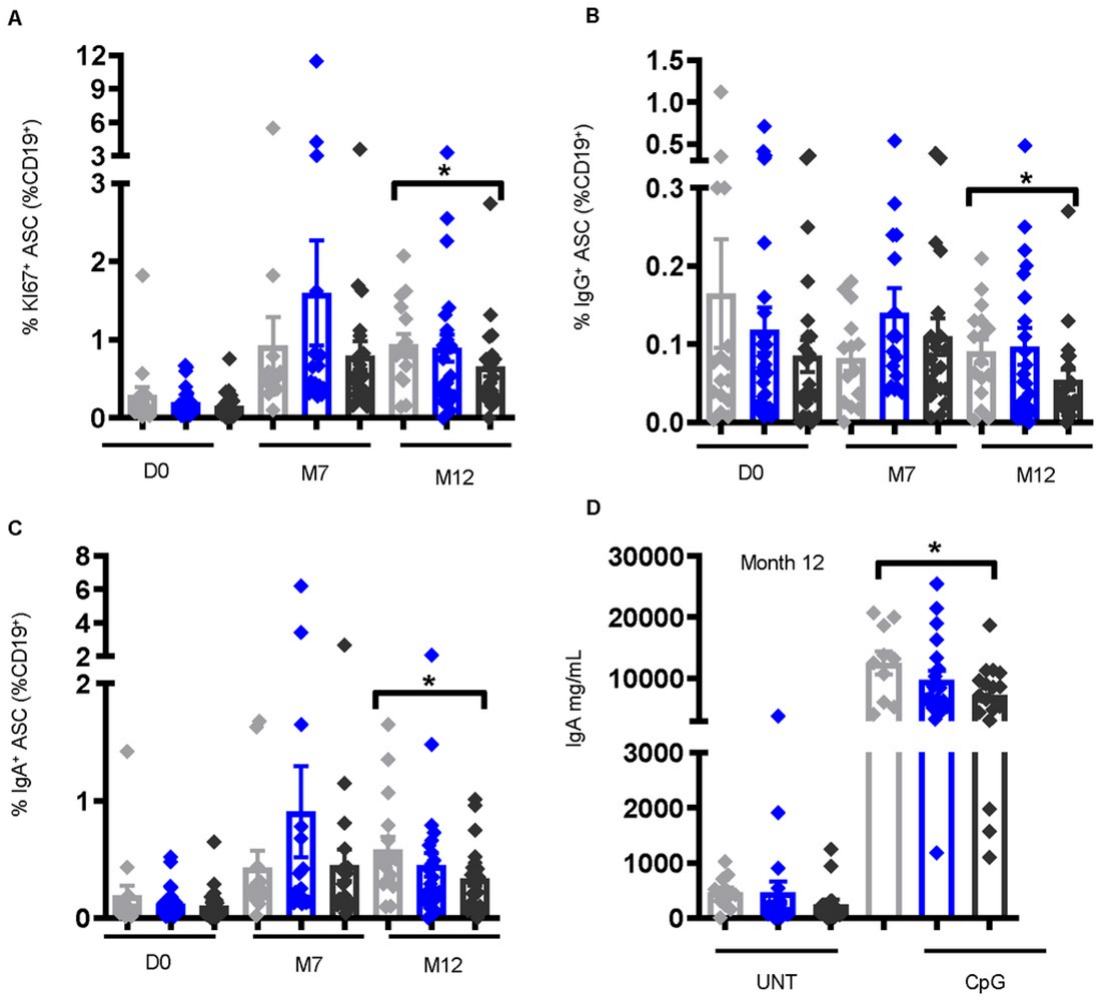
Frequencies of antibody secreting cells (ASCs) are lower at month 12 post-vaccination in individuals with high CAA concentration. Frequencies of (A) KI67^+^ ASCs [CD19^+^CD10^-^IgD^-^CD71^+^CD38^+^CD20^-^], (B) IgG^+^ ASCs, and (C) IgA^+^ ASCs, identified by flow cytometry of PBMCs pre-vaccination (D0) [non-infected, n = 16, low CAA, n = 29, high CAA, n = 24], M7 post-vaccination [non-infected, n = 14, low CAA, n = 17, high CAA, n = 20], and M12 post-vaccination [non-infected, n = 16, low CAA, n = 23, high CAA, n = 21]. Data shown as ± SEM. * P ≤ 0.05. Wilcoxon rank-sum test performed on non-infected vs. low CAA, or non-infected vs. high CAA, or low CAA vs. high CAA for each time point separately D0, M7, or M12. (D) IgA levels at M12 post-vaccination in the culture supernatant of PBMCs stimulated for 7 days with CpG (CpG-ODN [TLR9]) or UNT (untreated) [D0 non-infected, n = 14, low CAA, n = 27, high CAA, n = 21; M12 post-vaccination non-infected, n = 10, low CAA, n = 20, and high CAA, n = 17]. Data shown as ± SEM. * P ≤ 0.05. Wilcoxon matched-pairs signed rank test performed on UNT vs. CpG for each non-infected, or low CAA, or high CAA, and within the CpG-treated group a Wilcoxon rank-sum test on non-infected vs. low CAA, or non-infected vs. high CAA, or low CAA vs. high CAA. Non-infected- light grey, low CAA—blue, and high CAA—dark grey.

Cellular changes were further examined in response to *S*. *mansoni* infection and HepB vaccination by looking at the frequencies of activated B cells (ABC) and antigen-specific antibody-secreting B cells (ASC) [45] (S3 Fig). Frequencies of ABCs (no vs. high P = 0.048) and IgG^+^ABCs (no vs. high P = 0.015) were found to be significantly higher pre-vaccination in individuals with high CAA, with similar results at M7 (ABC, no vs. high P = 0.039; IgG^+^ABCs, no vs. high P = 0.032) and M12 (ABC, no vs. high P = 0.040; IgG^+^ABCs, no vs. high P = 0.033) post-vaccination (S4A and S4B Fig), indicative of a hyperactivated inflammatory B cell response [43]. Of importance and in sharp contrast, lower frequencies of KI67^+^ASC (no vs. high P = 0.040), IgG^+^ASC (no vs. high P = 0.046), and IgA^+^ASC (no vs. high P = 0.029) populations were observed in individuals with high CAA at M12 post-vaccination (Fig 5A–5C); and assessment of the correlation between ASCs and HepB titers showed a positive correlation between IgA^+^ASCs and HepB titers at M12 (t = 2.10, P = 0.041) (S4C Fig). B cell function was further investigated in the context of Ig class-switching by treating PBMCs from non-infected and *S*. *mansoni*-infected individuals with the TLR9 ligand CpG-ODN 2006 and analyzing the culture supernatant for Ig antibodies using a multiplex bead assay. Post-vaccination, IgA levels in the supernatant of CPG-stimulated PBMCs were found to be significantly lower in individuals with high CAA (no vs. high P = 0.031) (Fig 5D). These results imply that during vaccination, an elevated worm burden could influence humoral immunity, including the specific isotype induced, particularly by modulating changes in the B cell compartment resulting in a highly non-specific inflammatory B cell response, and a reduction in the vaccine-specific B cell response.

### Monocyte function is positively associated with effective Hepatitis B vaccine responses

**Fig 6:**
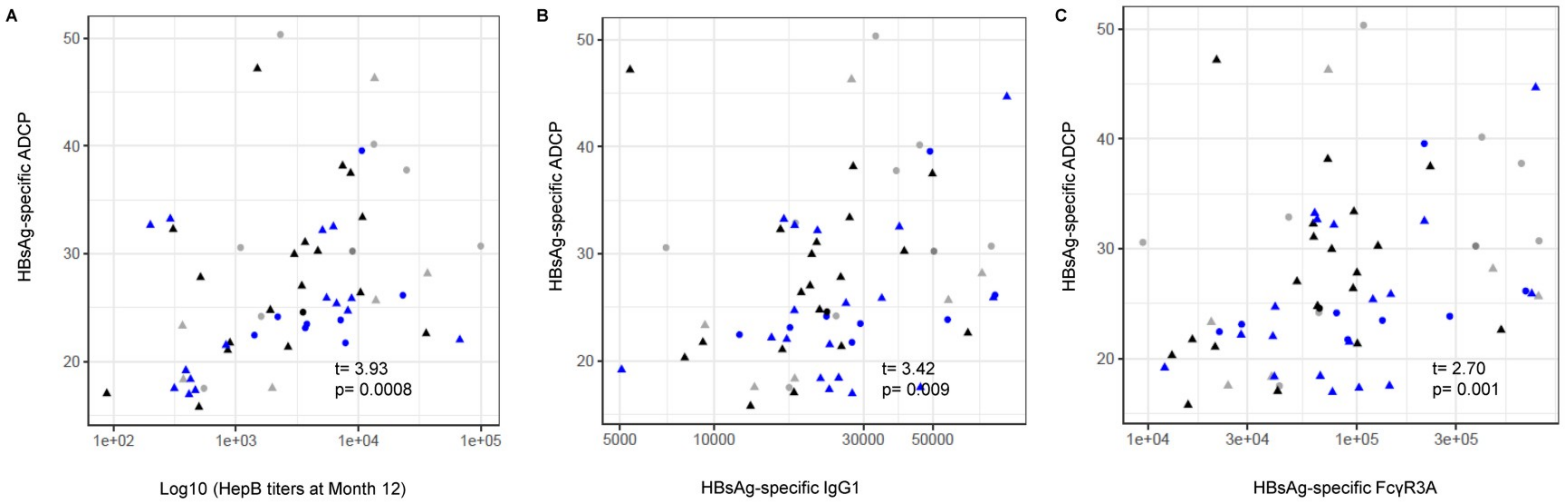
Monocyte function is important in and significantly correlate with Hepatitis B vaccine responses. Antibody-dependent cellular phagocytosis (ADCP) assays conducted with serum [pre-vaccination- non-infected, n = 14, low CAA, n = 20, high CAA, n = 18] and cells from the monocyte cell line THP-1. Scatter plots show HBsAg-specific ADCP associations with (A) Hepatitis B titers at M12, and with (B) HBsAg-specific IgG1, and (C) HbsAg-specific antibody binding to FcγR3A (CD16) expression determined by antibody subclass and Fc receptor binding assays. Spearman correlation and t-test were used to evaluate for the significance of the correlation. *coef* (regression coefficient). t (t-statistic). P ≤ 0.05 was considered significant. (Shape: triangle-Male, circle-Female; color: grey- non-infected, black- low CAA, blue- high CAA).

Our analysis in this study has pointed to the importance of key cytokines/chemokines involved in innate responses to infection detected pre-vaccination that may play a crucial role in modulating the adaptive immune response post-vaccination in *S*. *mansoni*-infected individuals with various levels of worm burden. To additionally investigate the importance of innate responses, the relationship between monocyte function and HepB vaccine responses was explored. Antibody-dependent cellular phagocytosis (ADCP) assays were conducted using the monocyte cell line THP-1, and ADCP was found to positively correlate with HepB titers at M12 (P = 0.0008) (Fig 6A), HepB surface antigen (HbsAg)-specific IgG1 titers (P = 0.009) (Fig 6B), and binding of HbsAg-specific antibodies to the cytotoxic Fc gamma receptor 3A (FcγR3A) (P = 0.001) (Fig 6C), indicating the importance of monocyte function in an effective humoral immune response to the HepB vaccine. As we did not observe any associations between monocyte subsets and CAA concentration, suggesting that monocyte frequency is not directly affected by *S*. *mansoni* infection, we were interested to further examine associations of innate immune function with CAA concentration by exploring the induction of cytokine/chemokines.

### Innate immune-related cytokines/chemokines are significantly lower pre-vaccination and day 12 post-vaccination in instances of high CAA concentration after TLR7/8 stimulation

**Fig 7:**
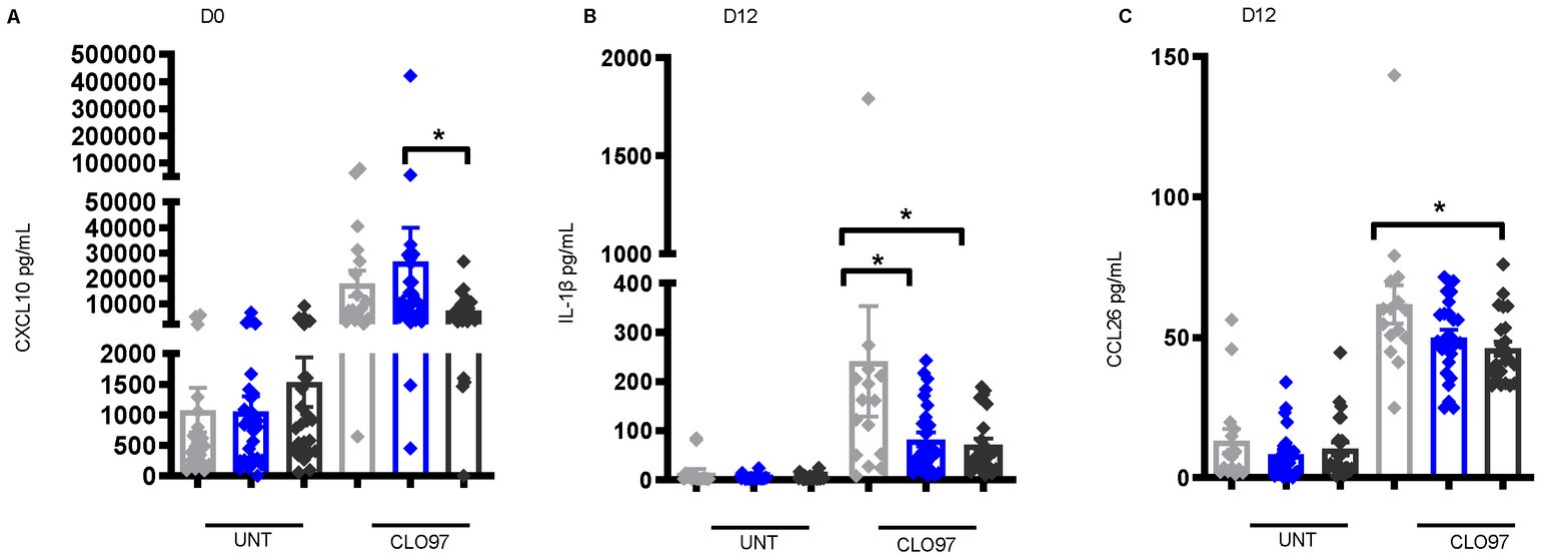
Lower levels of cytokines/chemokines important in innate immune cell function in *S*. *mansoni*-infected individuals pre-vaccination and day 12 post-vaccination. (A) CXCL10 levels pre-vaccination (D0) [non-infected, n = 19, low CAA, n = 31, and high CAA, n = 25], and (B) IL-1β, and (C) CCL26levels at D12 post vaccination [non-infected, n = 15, low CAA, n = 26, high CAA, n = 23] in the culture supernatant of PBMCs stimulated for 18 hours with CLO97 (TLR7/8 agonist) or untreated (UNT). Data shown as ± SEM. * P ≤ 0.05 Wilcoxon matched-pairs signed rank test performed on UNT vs. CLO97 or each non-infected, or low CAA, or high CAA, and within the CLO97-treated group a Wilcoxon rank-sum test on non-infected vs. low CAA, or non-infected vs. high CAA, or low CAA vs. high CAA. Non-infected- light grey, low CAA—blue, and high CAA—dark grey.

To investigate the importance of innate immune function in shaping the response to Hepatitis B vaccination during *S*. *mansoni* infection, PBMCs from non-infected and *S*. *mansoni*-infected individuals pre-vaccination and D12 post-vaccination were stimulated with a viral toll-like receptor (TLR) 7/8 agonist- imidazoquinoline compound CLO97, and the supernatant analyzed by multiplex bead assay for cytokines/chemokines after 18 hours of stimulation. Levels of the CXC chemokine IFN-γ-inducible protein 10 (CXCL10; IP-10), which plays a crucial role in activating innate immune cells, promoting dendritic cell (DC) maturation, and inducing protective T cell responses [46,47], were significantly lower in CLO97-treated supernatant from the high CAA group pre-vaccination (low vs. high P = 0.031) (Fig 7A). Additionally, supernatant levels of IL-1β (no vs. high P = 0.028) and CCL26 (no vs. high P = 0.014) were significantly lower in the high CAA group after treatment with CLO97 D12 post-vaccination (Fig 7B and 7C). Collectively, these cytokines/chemokines play important roles in early immune responses to viral infection and vaccination including modulation of inflammatory responses and adaptive cell recruitment, activation, and regulation [46–49]. These results indicate an association between *S*. *mansoni* infection and worm burden in the altering of the pre-vaccination, and early post-vaccination innate cytokine environment.

## Discussion

Vaccination is an essential tool in controlling the spread of infectious diseases, but variations of host immune responses to vaccines across populations (endemic infections, pre-existing infections, geography, biological sex, race/ethnicity) impact the generation of protective immune responses. These variations in vaccine responses, seen in communities with endemic diseases such as schistosomiasis, have recently been the topic of intense investigation [3,4]. We show that the pre-vaccination CAA values aligned with groups categorized by low and high worm burden displayed a bimodal distribution, and that increasing CAA concentration was negatively associated with vaccine-specific antibody levels at month 7, but not month 12. This was an interesting finding and served as the premise for further investigation to delineate what other molecules and cell populations might be at play, and responsible for these changes in vaccine-related immune responses post-vaccination. We observed that *Schistosoma*-specific responses pre-vaccination that were elevated in high CAA individuals (S5A Fig), were also elevated at month 12-post vaccination in those individuals (S5B Fig), indicating that in this community setting, the chances of re-infection and ineffective *Schistosoma* treatment is high and very possible, and the reason why understanding immune responses to vaccination is so important for these groups of individuals.

**Fig 8:**
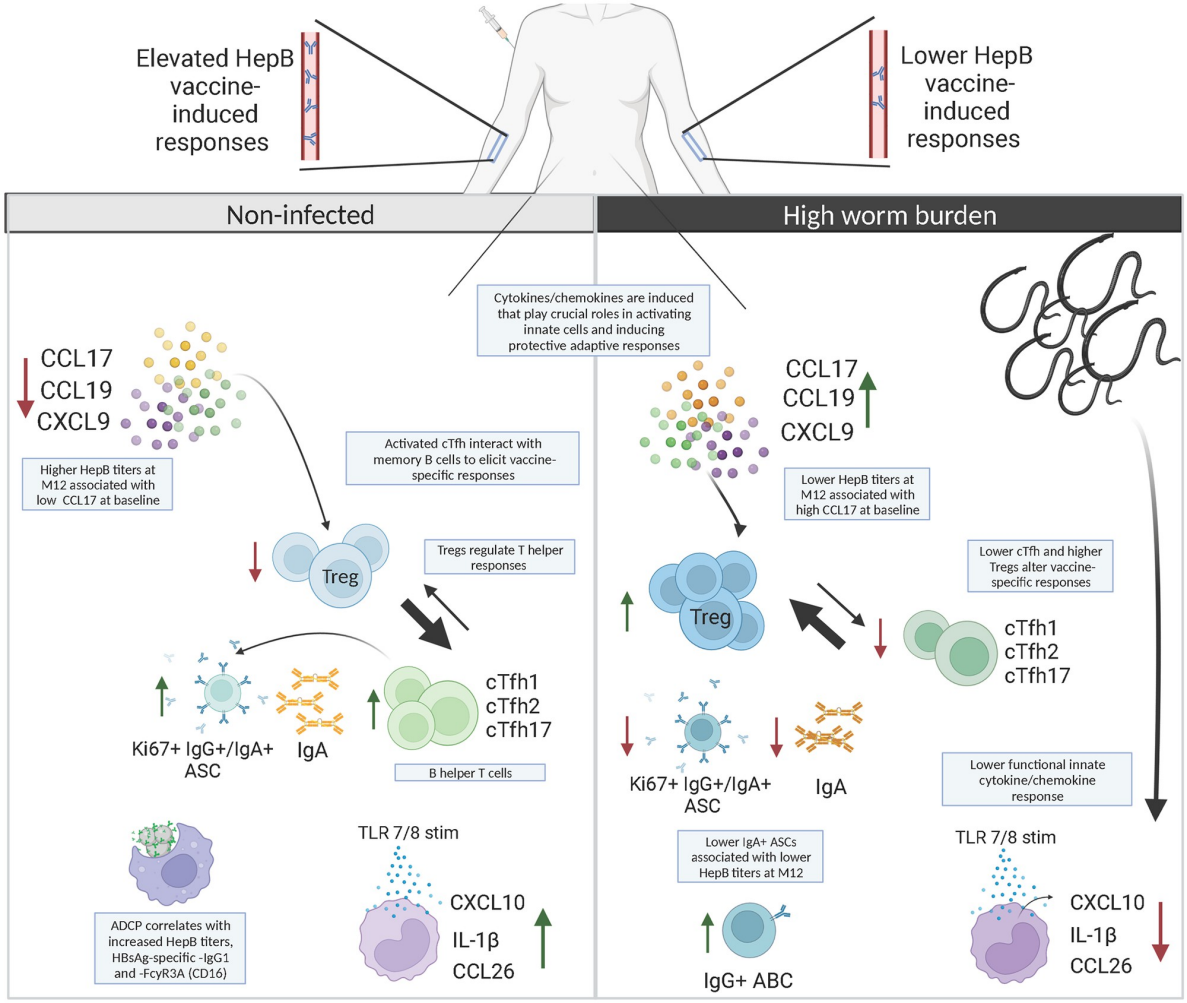
Immunological alterations in high worm burden Hepatitis B-vaccinated hosts with *Schistosoma mansoni* infection. In instances of high schistosome-specific circulating anodic antigen (CAA) concentration pre-vaccination (higher worm burden) we observe an association with altered HepB vaccine responses. High worm burden pre-vaccination is associated with higher levels of CCL17 and higher frequencies of Tregs post-vaccination. Concurrently, high worm burden is also associated with lower frequencies of the B-helper T cells, cTfh, lower proliferating and multi-isotype ASCs post-vaccination, and alterations in the early innate cytokine/chemokine microenvironment. These findings suggest that the level of *Schistosoma* worm burden in infected individuals can create a pre-vaccination microenvironment that cooperates to modulate optimal host immune responses to HepB vaccination thereby increasing the risk for endemic communities of infection against vaccine-preventable diseases. Created with Biorender.com.

The major strength of this study lies in this unique cohort, which allowed analysis of individuals pre-vaccination and post-vaccination, with different concentrations of CAA and likely different levels of worm burden from direct exposure, or no *S*. *mansoni* infection at all; and allowed us to comment on the impact of baseline *S*. *mansoni* infection on vaccine-induced responses, and more specifically if these induced responses were affected by worm burden. Our analysis of vaccine-specific antibody titers, cytokines/chemokines, and immune and adaptive cellular responses also allowed us to identify the links between high *S*. *mansoni* worm burden and lower HepB vaccine responses. While the outcomes of this present study were based solely on the influence of *S*. *mansoni* infection on HepB vaccination, we believe the results have the potential to direct this and other immunization programs in schistosomiasis-impacted communities where repeated exposure is likely. We summarize our immunological findings in Fig 8 and believe that cooperation of the multiple soluble and cellular factors in the instance of high *Schistosoma* worm burden pre-vaccination, can impact vaccine-related immune responses and hence contribute to how protective the vaccine will be. Similar findings have also been published on the role of key genes for cytokines/chemokines such as *CXCL10* and IL-1 mediators, as predictors of vaccination responses when looking at associations between pre-vaccination endotypes and antibody responses [50].

Our analysis showed induction of several proinflammatory molecules involved in lymphocyte migration and activation, including CCL19, CXCL9, and CCL17 in individuals with high CAA pre-vaccination suggesting a coordinated T cell recruitment and activation response in instances of high worm burden. Higher levels of CCL17 showed negative associations with HepB titers in cases of high worm burden, suggesting that CCL17 may be a potential biomarker for inefficient antibody development. CCL17 has been shown to play a key role in recruiting CCR4-expressing T helper and regulatory cells during inflammation [51–54]. While T helper 1 and 2 responses are predominant in Schistosomiasis [15], these chronically skewed environments have been known to be antagonistic to Tfh-B cell interactions that are important for inducing antigen-specific antibody responses [19,20,42]. Furthermore, alterations in innate immune function in *Schistosoma* infection observed in our cohort may potentially play a role in shifts in these cytokine levels; and previous models have also shown that elevation of T-helper cytokines in *S*. *mansoni* infection correlate specifically with lower vaccine-specific responses to immunization against *Mycobacterium bovis* (BCG) [55], HepB, and tetanus toxoid [24].

Several Tfh cell subsets involved in antibody induction during infection and vaccination, including cTfh cells in the periphery, play a key role the generation of high-affinity antibody-producing plasma cells and memory B cells [41]. We also now know that changes observed in cTfh profiles reflect the cytokine microenvironment, and specific cTfh subpopulations are known to drive B cell class switching [56]. We demonstrated that individuals with high CAA had lower cTfh1 and -2 cells pre-vaccination, which was sustained post-vaccination for cTfh1 and cTfh17 cells in this group. More specifically, a positive association between HepB-specific memory CD4^+^ T cell responses and HepB-specific antibody production was observed. These data highlight the vital role of HepB-specific memory CD4^+^ T helper cells in driving antibody responses, and that changes in this compartment directly influences vaccine responses. The changes we observed pre- and post-vaccination in the cTfh1 subpopulation suggest that these cells could be a marker of protective immunity, as their increase in cases of vaccination against yellow fever correlated with increased neutralization antibody responses, and cTfh1 were found to efficiently induce memory B cells to produce influenza-specific IgG post-vaccination [57,58]. In parallel, we also reported higher frequencies of Foxp3^+^Tregs in individuals with high CAA post-vaccination, suggesting a polarization towards higher frequencies of Tregs. These data strongly suggest that in instances of high worm burden, robust regulatory responses can be induced by key pro-Treg cytokines pre-vaccination, such as CCL17 [53,59], and other cytokines such as IL-2, which we also observe to be elevated pre-vaccination (S6 Fig), that can inhibit Tfh responses [60]. We believe this environment at baseline can dictate varied changes to the T cell profile and kinetics post-vaccination and skew the ratio of Tregs: cTfh cells when there is a high worm burden.

In further support of our findings, Yin et al. recently reported that lower responders to HepB vaccination had lower frequencies of cTfh cells and antibody-secreting plasmablasts [44]; and Musaigwa and colleagues describe that *S*. *mansoni* infection specifically induces cell death in bone marrow plasmablasts and plasma cells [61]. We show in addition to a lower frequency of cTfh cells in individuals with high CAA, a significant increase in activated B cells but a reduction in proliferating (antigen-specific) ASCs and ASCs expressing IgG and IgA; and that IgA^+^ASCs positively correlate with HepB titers. In addition to these data, we see a significant reduction in TLR9-induced IgA levels post-vaccination. In combination, these results point to a direct role for high worm burden environments in influencing changes in the immune landscape polarizing it away from cTfh and B cell responses which would hamper vaccine-specific antibody production post-vaccination.

We show the importance of innate immune responses, where we observe that the levels of TLR7/8-induced cytokines varied with *S*. *mansoni* worm burden, evidenced by mixed responses pre- and 12 days post-vaccination. Lower CXCL10 (IP-10) levels in instances of high CAA pre-vaccination was an interesting finding, as this chemokine plays a crucial role in the activation and recruitment of leukocytes and monocytes, DC maturation, and induction of protective T and B cell responses [46,47]. These data are in line with other reports showing that a CXCL10– inclusive signature is associated with effective immune responses to a SARS-CoV-2 vaccine after the 1st vaccination [62], and that a link between CXCL10 levels in the serum and innate responses is associated with increased vaccine-specific antibody titers [63,64]. Our data, combined with other reports, suggest a role for CXCL10 in modulating the early response to vaccination.

Notably, it has been demonstrated that deworming can enhance the host immune response to vaccination [23], and some studies have shown that PZQ treatment can partially restore vaccine-induced immune responses [61,65]. While all enrolled individuals that were egg positive in stool samples in this current study were treated with PZQ at D12 post-vaccination (Fig 1), it is possible that *Schistosoma*-related effects on baseline immune responses may have already occurred. Furthermore, the rate of reinfection and the extent to which the initial infection was cleared is also unknown, particularly as we see elevated *Schistosoma*-specific responses at M12 (S5B Fig), and several proinflammatory cytokines at baseline remained elevated at month 12 post-vaccination. It is probable that individuals in this fishing community with a higher baseline CAA concentration are getting more reinfections with time, creating a cycle of high CAA concentration driving inefficient immune responses to secondary antigen. Despite these limitations, we still observed differences in vaccine-induced responses in individuals with varying pre-vaccinated levels of CAA, suggesting the baseline environment plays a role in dictating the immune response to vaccination. A similar observation was seen in an animal study of chronic schistosomiasis and HIV vaccination where schistosomiasis suppressed vaccine responses, and this was maintained regardless of anti-helminth treatment [66]. Overall, these results suggest that pre-vaccination levels of *Schistosoma* worm burden are associated with varying immunomodulatory changes that can influence a host’s response to unrelated antigen, such as those in vaccines.

In summary, we believe high worm burden in *S*. *mansoni* infection is associated with the dysregulation of vaccine-induced responses by the cooperation of key cytokines/chemokines that lead to elevated inflammation, and lower levels of cytokines that promote the frequency and function of innate and adaptive immune subsets important in generating vaccine-specific antibodies. The results from our study could be applied to instruct ongoing and future projects to benefit vaccination programs in *Schistosoma*- and other helminth- endemic communities, particularly in the design of studies that address specific hypotheses such as recently published study protocols from Nkurunungi and colleagues which describe the effect of a more intensive intervention with anti-helminth treatment on vaccine responses in adolescents in Uganda [67,68].

## Methods

### Ethics statement

Studies were granted approval by the Uganda Virus Research Institute Research Ethics Committee, reference number GC/127/15/07/439 and the Uganda National Council of Science and Technology, reference number HS 1850. Informed written consent was obtained from all participants prior to being enrolled in the studies.

### Hepatitis B vaccine study design

Healthy adult volunteers who were enrolled in this study were one-arm of a community-based prospective investigation that took place in fishing communities located in Entebbe, Uganda, called the Stimulated Vaccine Efficacy Trial (SiVET). SiVET was set up to identify populations and assess the implementation of efficacy trial procedures. HepB and typhoid vaccines were selected as simulated vaccines due to the potential benefit to the fishing communities. Participants aged 18 to 49 years, males and non-pregnant females were enrolled starting in 2015 from one mainland lakeshore fishing community and one island community along Lake Victoria, in Wakiso district. (S1 Table). Inclusion criteria encompassed HIV-uninfected, negative HepB surface antigen (HbsAg) and core antibody tests, capability, and willingness to provide written informed consent to receive HepB vaccine, and consent for follow-up leading up to 12 months after the first study immunization. Volunteers were not prescreened for active malaria and latent TB infections. Of the persons that enrolled for the first dose of the HepB vaccine, 75 completed the vaccination program and received all 3 doses. These donor samples were available to be used in this present sub study. Adults were screened up to 6 weeks before the first study injection of the Hep vaccine ENGERIX-B (GlaxoSmithKline Biologicals, derived from recombinant subunit Hepatitis B surface antigen adsorbed on aluminum hydroxide) followed by two booster doses given at months 1 and 6 (Fig 1). Doses were given intramuscularly in the deltoid muscle at 1mL volume containing 20 μg of the vaccine. Whole blood and plasma were collected pre-vaccination (D0), post-1^st^ injection (D3, D7, and D12), and post-booster injections on the day the infections were administered (M1 and M6) (Fig 1).

### Identification and treatment of *S*. *mansoni*-infected enrolled individuals

Stool samples were collected from enrollees pre-vaccination (D0), prior to the first dose of the HepB vaccine, and at D3 and D7 post-vaccination. Measurement was done chronologically with D0 specimen first, and if worms were not detected, D3 or and D7 specimen were tested accordingly. *Schistosoma* egg counts were subsequently conducted and if stool sample/s were positive, PZQ treatment was administered. Volunteers were treated at day 12 with Praziquantel (PZQ) as appropriate, for egg counts detected in stool samples at enrollment, D3, and/or D7 (Fig 1). *Schistosoma-*infected participants received 40mg/kg body weight single dose (average 2.4 g) of PZQ.

### Circulating anodic antigen (CAA) assay

After the completion of the vaccine study, frozen sera samples were analyzed by a circulating anodic antigen assay (CAA). Analysis was carried out on pre-vaccination (Day 0) on serum samples from enrolled participants to reveal active *Schistosoma* infection using published techniques [29–31,34]. Human negative serum (Sanquin, blood donors, the Netherlands) was spiked with a known concentration of CAA and dilutions made up to eight standard points to provide an appropriate standard series. An extra negative serum sample was included as a duplicate negative control. Standards of known concentrations of CAA and serum negative controls were used to create a calibration curve to quantify CAA levels and cutoffs. Samples were evaluated using the SCAA500 protocol with a lower limit of detection threshold of 3pg/mL; 500μL serum and standards were extracted with an equal volume of 4% w/v trichloroacetic acid (TCA; Merck Life Science NV, the Netherlands), vortexed and incubated at ambient temperature for five minutes. Thereafter, samples and standards were briefly vortexed and spun at 13000g for five minutes and 0.5 mL of clear supernatant was concentrated to 20 mL using Amicon Ultra-0.5 Centrifugal Filter Units devices with a molecular weight cut-off of 10 kDa (Merck Life Science N.V., the Netherlands). Note, for samples with insufficient serum volumes the SCAA20 test format was used which requires only 20uL serum and no concentration step but has a lower limit of detection threshold of 30pg/mL. The resulting TCA soluble fraction (20μL) was added to wells containing 100 ng dry UCP particles [69] (400 nm Y_2_O_2_S:Yb^3+^,Er^3+^) coated with mouse monoclonal anti-CAA antibodies [29] hydrated with 100 mL of high salt lateral flow buffer (HSFS: 200 mM Tris pH8, 270 mM NaCl, 0.5% (v/v) Tween-20, 1% (w/v) BSA. After being incubated for one hour at 37°C while shaking at 900rpm the CAA lateral flow strips (32) were placed in the wells, and samples allowed to flow. The strips were then dried overnight and analyzed using an Upcon reader (Labrox Oy, Turku, Finland). The test line signals (T; relative fluorescent units, peak area) were normalized to the flow control signals (FC) of the individual strips and the results expressed as Ratio value (R = T/FC).

### Whole blood processing and sample storage

PBMCs isolation/storage: Whole Blood collected in NaHeparin tube (BD, NJ, USA) was layered over 20ml of Histopaque (Sigma-Aldrich, Darmstadt, Germany) and centrifuged at 400g for 40 minutes at room temperature with no centrifuge brakes. Lymphocytes, platelets, and monocytes found at the plasma-separating medium interface (buffy coat) were recovered and washed in Hanks Balanced Salt solution to remove contaminating platelets, separation media and plasma. The resulting PBMCs (lymphocytes and monocytes) were resuspended in complete RPMI media (RPMI 1640 media supplemented with 10% Fetal Bovine Serum (FBS), 10mM Hepes buffer, 2mM L-glutamine, 1mM sodium pyruvate and 1X penicillin-streptomycin) for counting and concentrated at 10million cells/mL/ vial in FBS with 10% DMSO, frozen down using a rate-controlled freezer and stored in liquid nitrogen.

### Serum separation

Specific vacutainers for serum (SST, Plymouth UK) were inverted twice to mix blood and then centrifuged at 1200 g for 10 minutes at room temperature with maximum acceleration and brake after which aliquots into pre-labeled cryovials were made and stored at below -70°C.

### Plasma separation

Plasma recovered from the PBMC separation tube was centrifuged at 1800 g for 20 minutes at room temperature and the maximum value for acceleration and brake, after which samples were aliquoted into pre-labeled cryovials and stored at at -70°C.

### Hepatitis B antibody testing

Each serum sample was assayed at screening using three different HBV infection tests for: Hepatitis B surface antigen (HbsAg), Hepatitis B core antibody (anti-HBc), and Hepatitis B surface antibody (anti-HBs). The HbsAg and antiHBc antibodies were tested for using the VIDAS HbsAg Ultra and VIDAS anti-HBc Total II (Biomerieux SA, France) kits respectively on the MinVidas analyzer. The anti-HBs testing was done using the Cobas e 411 analyzer (Roche Diagnostics, Mannheim, Germany). The anti-HBs titer cut off value was 10IU/L. All three tests were used only at screening to differentiate between possible past exposure from active infection, and to ensure only HepB-negative individuals were recruited and administered the vaccine. Measurement of anti-HBs antibody was conducted for all subsequent follow-up visits.

### PBMC culture

PBMCs from non-infected and *S*. *mansoni*-infected individuals pre-vaccination and post-vaccination were thawed and rested for 3 hours before being placed into culture. Samples were used for ex-vivo immunostaining and flow cytometry analysis, and for TLR stimulation assays. PBMCs were suspended in RPMI medium supplemented with L-glutamine (Corning Cellgro, Manassas, VA, USA), 10% FBS and 1 X [50 U] penicillin-streptomycin (Invitrogen, Carlsbad, CA, USA) (R10F). 1 x10^6^ cells were added to 5-ml polypropylene tubes for CLO97 stimulation (Imidazoquinoline Compound; TLR7/8 agonist) (Invivogen, San Diego, CA, USA), and 0.5 x 10^6^ cells added to the wells of 96-well U-bottom plates for CpG-ODN 2006 stimulation (TLR9 agonist) (Invivogen). Optimal concentration for CLO97 stimulation was selected as previously described [70]. CpG-ODN 2006 was titrated on CFSE-labeled PBMCs, and optimal TLR9 activity assessed on proliferating CD19^+^ cells by flow cytometry. Cells were allowed to rest at 37°C under a 5% CO^2^ atmosphere for 3 hours prior to addition of TLR agonists at the following concentrations: CLO97 (0.5ug/ml) and CpG (1ug/ml). Cells were cultured also at 37°C under a 5% CO^2^ atmosphere for 18hrs (CLO97), and 7 days (CpG-ODN 2006). After culture, PBMCs were collected and washed in preparation for analysis by multiplex immunoassays or flow cytometry. For HBV peptide stimulation, PBMCs were first labeled with CFSE using a Cell Trace CFSE cell proliferation kit (ThermoFisher Scientific, Waltham, MA, USA), and rested for 3 hours in the conditions described above. In a 96-well deep well plate (USA Scientific, Ocala, FL, USA), 2 x 10^6^ cells in 1mL RPMI medium supplemented with 8% human serum (Access Biologicals, Vista, CA, USA), 1% penicillin-streptomycin and 10ng/mL IL-2 (Miltenyi Biotec, Auburn, CA, USA) (R8H-IL-2) were stimulated with either Pepmix HBV (Large envelope protein) (JPT Peptide Technologies, Berlin, Germany) at 1ug/mL or control (R8H-IL-2 with 0.2% DMSO) on Day 1 and cultured in the conditions described above. On Day 3, cells were supplemented with fresh R8H-IL-2 by half media replacement; and on Day 6, PBMCs were collected and washed in preparation for measurement of immune recall responses by flow cytometry.

### Cytokine and chemokine analysis (multiplex immunoassay)

Plasma collected from whole blood and supernatant collected from stimulated PBMCs were analyzed for chemokines/cytokines and Ig isotypes using magnetic bead multiplex assays. The following human analyte premixed panels were used: Bio-Plex human chemokine panel (Bio-Rad, Hercules, CA, USA): I-309 (CCL1), MCP-1 (CCL2), MIP-1α (CCL3), MCP-3 (CCL7), MCP-2 (CCL8), Eotaxin (CCL11), MCP-4 (CCL13), MIP-1α (CCL15), TARC (CCL17), MIP-3β (CCL19), 6Ckine (CCL21), MIP-3α (CCL20), MDC (CCL22), MPIF-1 (CCL23), Eotaxin-2 (CCL24), TECK (CCL25), Eotaxin-3 (CCL26), CTACK (CCL27), GM-CSF, GRO-α (CXCL1), GRO-β (CXCL2), ENA-78 (CXCL5), GCP-2 (CXCL6), MIG (CXCL9), IP-10 (CXCL10), I-TAC (CXCL11), SDF-1A+β (CXCL12), BCA-1 (CXCL13), SCYB16 (CXCL16), Fractalkine (CX3CL1), MIF, IL-1β, IL-2, IL-4, IL-6, IL-8, IL-10, IL-16, TNF-α, and IFN-γ. Custom ProcartaPlex 34-plex (ThermoFisher Scientific): APRIL, BAFF, CD30, CD40L, G-CSF, IFN-A, IL-12P70, IL-13, IL-15, IL-16, IL-17A, IL-18, IL-1A, IL-20, IL-21, IL-22, IL-23, IL-27, IL-2R, IL-3, IL-31, IL-5, IL-7, IL-9, LIF, M-CSF, TNF-R2, TNF-B, TRAIL, TSLP, TWEAK, VEGF-A, IFNB, and IL-29/IFN-lambda1. Bio-Plex human isotype panel (Bio-Rad): IgM, IgA, IgG1, IgG2, IgG3, IgG4. IgE was measured using Bio-Plex human IgE isotype assay (Bio-Rad). The manufacturer’s protocol was followed. Data was acquired on a Bio-Plex 200 system (using bead regions defined in the protocol) and analyzed with the Bio-Plex Manager 6.1 software (Bio-Rad).

### Flow cytometry analysis of PBMCs

For ex-vivo analysis, 1 x 10^6^ PBMCs per well were incubated with Fixable aqua viability stain 405 (ThermoFisher Scientific) to discriminate dead from live cells then stained with antibody panels described (S2 and S3 Tables). To detect intracellular expression of markers, cells were fixed and permeabilized using the Foxp3/Transcription factor staining buffer set (ThermoFisher Scientific) and immunostained using FOXP3-FITC (clone, PCH101) (ThermoFisher Scientific) (S2 Table) and KI67-FITC (BD Biosciences, San Jose, CA, USA) (S3 Table). Following HBV peptide stimulation, PBMCs were stained with the surface antibody panel described (S4 Table), then stained with 7-AAD (BD Biosciences) for 10 minutes prior to analysis to discriminate dead from live cells. Flow cytometry measurements were made on a BD LSRFortessa (BD Biosciences) and collected data analyzed using FlowJo software version 10.7.1 (BD).

### Antibody-dependent cellular phagocytosis (ADCP) assay

An antibody-dependent cellular phagocytosis (ADCP) assay was conducted to investigate monocyte function. HbsAg was biotinylated using an EZ-Link Sulfo-NHS-LC-Biotinylation Kit (ThermoFisher Scientific) and coupled to FluoSpheres NeutrAvidin-Labeled Microspheres (ThermoFisher Scientific). To form immune complexes, antigen-coupled beads were incubated for 2 hours at 37°C with 1:25 diluted serum samples and then washed to remove unbound immunoglobulin. After washing, immune complexes were incubated for 16–18 hours with a monocyte cell line, THP-1 cells [25,000 THP-1 cells per well at a concentration of 1.25×105 cells/ml in RPMI (ThermoFisher Scientific) + 10% FBS (Sigma-Aldrich), and following incubation, cells were fixed with 4% paraformaldehyde (Poly Scientific R&D, Bay Shore, NY, USA). The percentage of fluorosphere positive cells was analyzed on a LSRII flow cytometer (BD Biosciences). The phagocytosis score was defined.

### Antibody subclass and Fc receptor binding

HbSAg-specific antibody subclass titers and Fc receptor binding profiles were analyzed with a custom multiplex Luminex assay as described previously [71]. In brief, HbSAg was coupled to magnetic Luminex beads (Luminex Corp, TX, USA). Coupled beads were incubated with diluted serum samples, washed, and IgG subclasses detected with a 1:100 diluted PE-conjugated secondary antibody for IgG1 (clone: HP6001; Southern Biotech, AL, USA). For the FcγR binding, a respective PE–streptavidin (Agilent Technologies, Santa Clara, CA, USA) coupled recombinant and biotinylated human FcγR protein was used as a secondary probe.

### Statistics

Statistical inference evaluating the differences of HepB titers between non-infected versus low CAA, non-infected versus high CAA and low CAA versus high CAA was determined using Wilcoxon rank-sum test. Wilcoxon rank-sum tests were performed on non-infected vs. low CAA, or non-infected vs. high CAA, or low CAA vs. high CAA within each time point for plasma Luminex assays, and ex-vivo flow cytometry analysis. Pairwise Wilcoxon signed rank test were performed on untreated vs. treated for each non-infected, or low CAA, or high CAA for CpG, HBV-LEP and CLO97 stimulation assays; and within the treated groups, a Wilcoxon rank-sum test was conducted on non-infected vs. low CAA, or non-infected vs. high CAA, or low CAA vs. high CAA. Linear regression analyses were adjusted for sex (no significant association between age and HepB titers) and student t-tests were used to evaluate for the significance of the association. Sensitivity analyses were performed using the R package sensemakr [72] used to assess the robustness of the results of the linear regression analysis to an unobserved confounding variable. Benjamini-Hochberg adjustment was used to control for multiple testing for each assay. P ≤ 0.05 was considered significant for all analyses.

## Supporting information

S1 Fig Flow cytometry gating strategy for T cell populations. Representative plots showing non-circulating T follicular helper cells (cTfh) populations as CD3^+^CD4^+^CD45RA^-^CD25^-^CXCR5^-^ and cTfh populations as CD3^+^CD4^+^CD45RA^-^CD25^-^CXCR5^+^, identifying cTfh1 as CXCR3^+^, cTfh2 as CXCR3^-^CCR6^-^, and cTfh17 as CXCR3^-^CCR6^+^. Regulatory T cells (Tregs) were identified as CD3^+^CD4^+^CD45RA^-^CD127^-^CD25^+^Foxp3^+^.
(TIF)

S2 Fig Frequencies of non-circulating T follicular helper cells pre- and post-vaccination. Frequencies of non cTfh [CD3^+^CD4^+^CD45RA^-^CD25^-^CXCR5^-^] were identified by flow cytometry of PBMCs from individuals pre-vaccination (D0) non-infected, n = 19, low CAA, n = 31, and high CAA, n = 25, M7 post-vaccination non-infected, n = 14, low CAA, n = 17, and high CAA, n = 20, and M12 post-vaccination non-infected, n = 16, low CAA, n = 24, and high CAA, n = 20. Data shown as ± SEM. Non-infected- light grey, low CAA—blue, and high CAA—dark grey.
(TIF)

S3 Fig Flow cytometry gating strategy for B cell populations. Representative plots showing activated B cells (ABC) as CD19^+^CD10^-^IgD^-^CD71^+^CD38^-^CD20^+^ and antibody secreting B cells (ASC) as CD19^+^CD10^-^IgD^-^CD71^+^CD38^+^CD20^-^.
(TIF)

S4 Fig Frequencies of Activated B cells (ABC) were elevated in *S*. *mansoni* infection pre- and post-vaccination. Frequencies of (A) ABCs [CD19^+^CD10^-^IgD^-^CD71^+^CD38^-^CD20^+^], and (B) IgG^+^ ABCs, were identified by flow cytometry of PBMCs pre-vaccination (D0) [non-infected, n = 16, low CAA, n = 29, high CAA, n = 24], M7 post-vaccination [non-infected, n = 14, low CAA, n = 17, high CAA, n = 20], and M12 post-vaccination [non-infected, n = 16, low CAA, n = 23, high CAA, n = 21]. Data shown as ± SEM. * P ≤ 0.05. Wilcoxon rank-sum test performed on non-infected vs low CAA, or non-infected vs high CAA, or low CAA vs high CAA for each time point separately D0, M7, or M12. Non-infected- light grey, low CAA—blue, and high CAA—dark grey. (C) Linear regressions fit between Hepatitis B titers and IgA^+^ ASCs, adjusted for sex, and student t-tests evaluated for the significance of the association. t (t-statistic). P ≤ 0.05 was considered significant. (Shape: triangle-Male, circle-Female; color: grey- non-infected, black- low CAA, blue- high CAA).
(TIF)

S5 Fig Elevated expression of *Schistosoma*-specific IgG4 pre- and post- vaccination. The mean Florescence intensity (MFI) of serum *S*. *mansoni*-specific IgG4 at (A) D0 [non-infected no, n = 15, low CAA, n = 21, and high CAA, n = 20], (B) M12 post-vaccination [non-infected no, n = 14, low CAA, n = 26, and high CAA, n = 20]. A student t-test was used to evaluate for the significance of the correlation. P ≤ 0.05 was considered significant.
(TIF)

S6 Fig Elevated plasma IL-2 in *S*. *mansoni*-infected individuals pre-vaccination. IL-2 levels in the plasma of non-infected, n = 19, low CAA, n = 32, and high CAA, n = 24 individuals pre-vaccination (day 0). Data shown as ± SEM. * P ≤ 0.05. Wilcoxon rank-sum test performed on non-infected vs low CAA, or non-infected vs high CAA, or low CAA vs high CAA for each time point separately. Non-infected- light grey, low CAA—blue, and high CAA—dark grey.
(TIF)

S1 Table Study participant information. (DOCX)

S2 Table Ex vivo T cell staining panel for PBMCs. (DOCX)

S3 Table Ex vivo B cell staining panel for PBMCs. (DOCX)

S4 Table T cell staining panel for PBMCs after Hepatitis B peptide stimulation. (DOCX)
