# Supplementary figures and images for: Schistosoma mansoni infection alters the host pre-vaccination environment resulting in blunted Hepatitis B vaccination immune responses
Source: PLoS Negl Trop Dis. 2023 Jul 5;17(7):e0011089. doi: 10.1371/journal.pntd.0011089 (PMC10351710; doi:10.1371/journal.pntd.0011089)

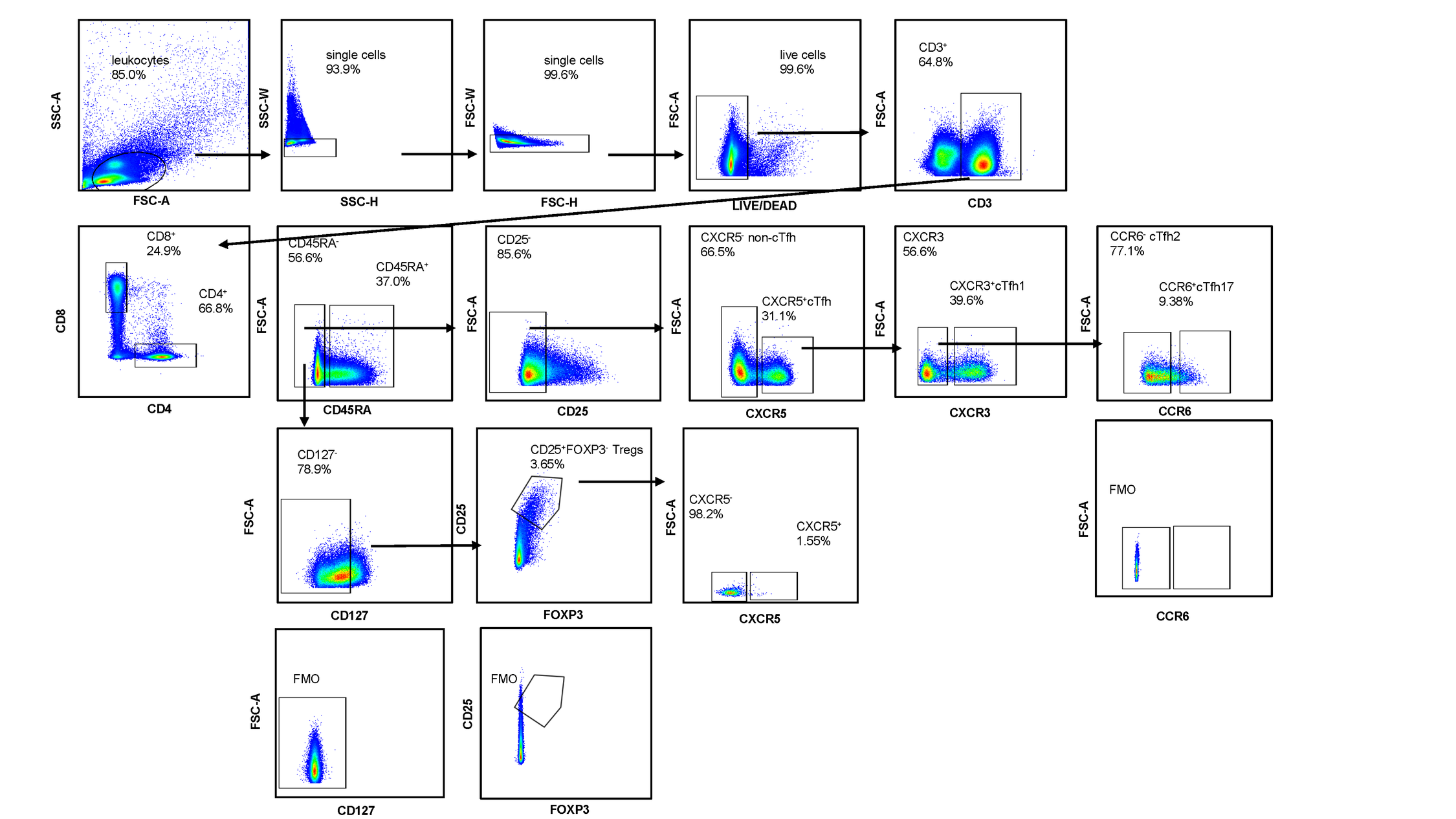

Supplement: S1 Fig — Representative plots showing non-circulating T follicular helper cells (cTfh) populations as CD3+CD4+CD45RA-CD25-CXCR5- and cTfh populations as CD3+CD4+CD45RA-CD25-CXCR5+, identifying cTfh1 as CXCR3+, cTfh2 as CXCR3-CCR6-, and cTfh17 as CXCR3-CCR6+. Regulatory T cells (Tregs) were identified as CD3+CD4+CD45RA-CD127-CD25+Foxp3+. (TIF) [file pntd.0011089.s001.tif]

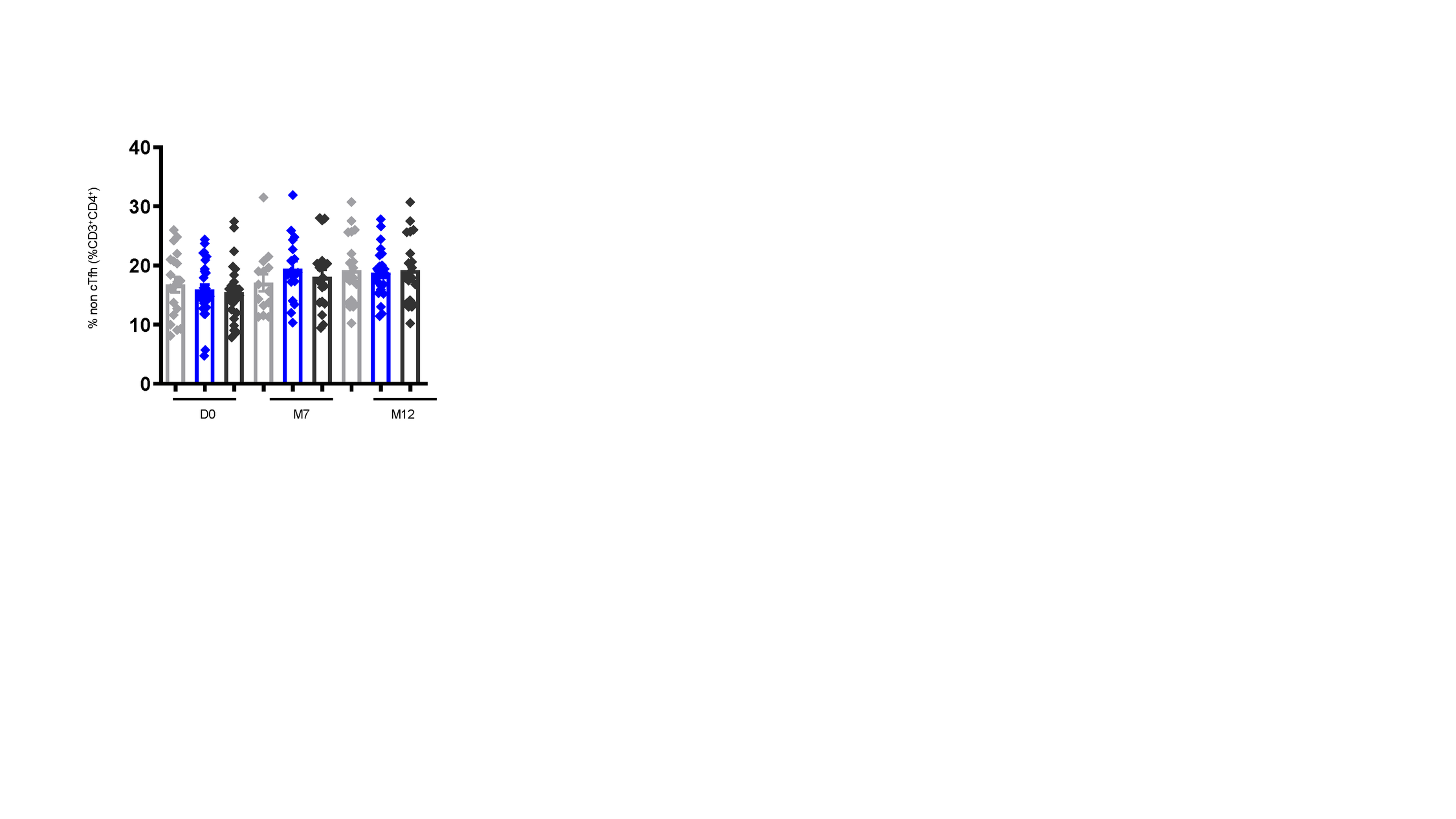

Supplement: S2 Fig — Frequencies of non cTfh [CD3+CD4+CD45RA-CD25-CXCR5-] were identified by flow cytometry of PBMCs from individuals pre-vaccination (D0) non-infected, n = 19, low CAA, n = 31, and high CAA, n = 25, M7 post-vaccination non-infected, n = 14, low CAA, n = 17, and high CAA, n = 20, and M12 post-vaccination non-infected, n = 16, low CAA, n = 24, and high CAA, n = 20. Data shown as ± SEM. Non-infected- light grey, low CAA—blue, and high CAA—dark grey. (TIF) [file pntd.0011089.s002.tif]

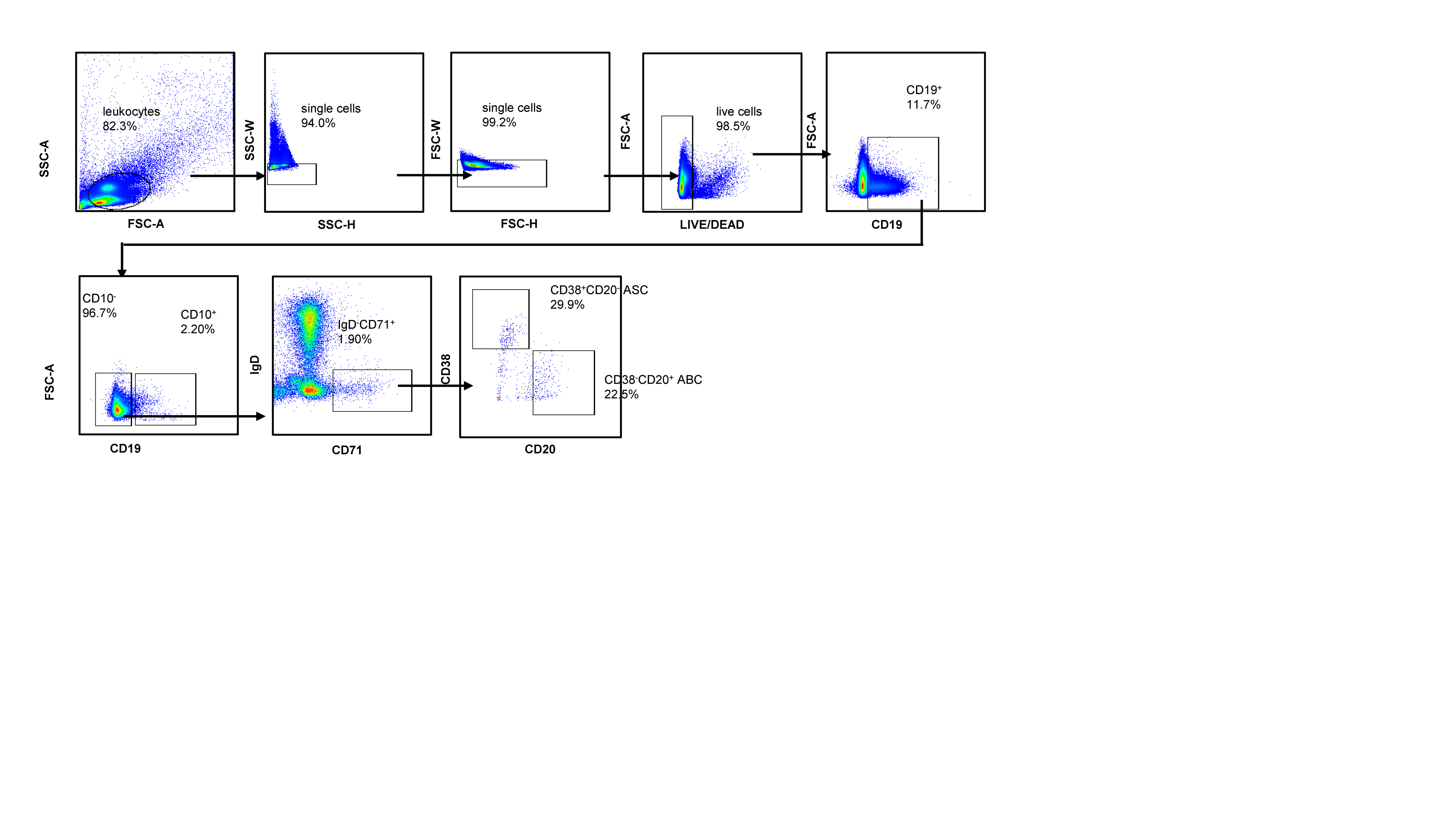

Supplement: S3 Fig — Representative plots showing activated B cells (ABC) as CD19+CD10-IgD-CD71+CD38-CD20+ and antibody secreting B cells (ASC) as CD19+CD10-IgD-CD71+CD38+CD20-. (TIF) [file pntd.0011089.s003.tif]

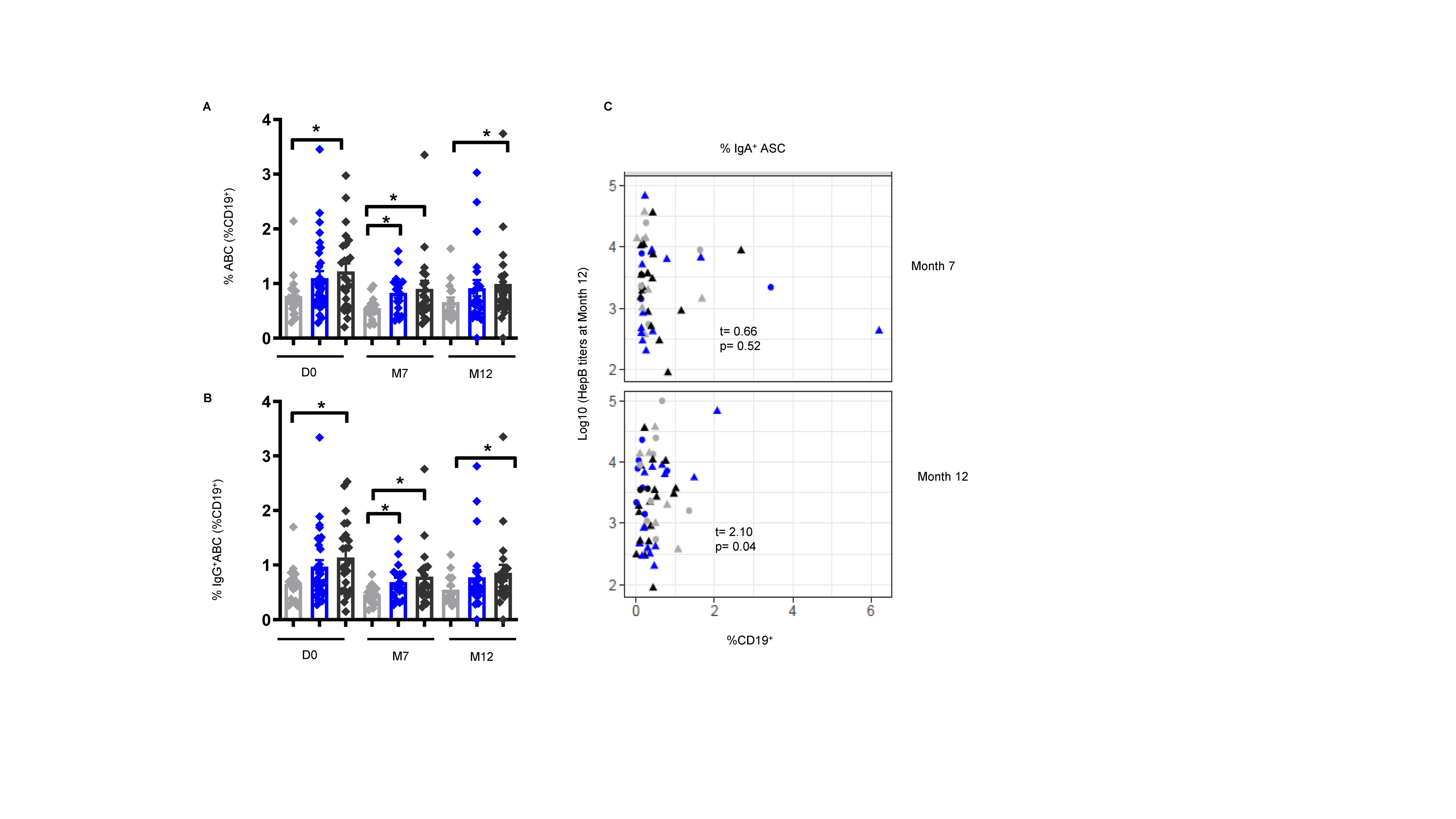

Supplement: S4 Fig — Frequencies of (A) ABCs [CD19+CD10-IgD-CD71+CD38-CD20+], and (B) IgG+ ABCs, were identified by flow cytometry of PBMCs pre-vaccination (D0) [non-infected, n = 16, low CAA, n = 29, high CAA, n = 24], M7 post-vaccination [non-infected, n = 14, low CAA, n = 17, high CAA, n = 20], and M12 post-vaccination [non-infected, n = 16, low CAA, n = 23, high CAA, n = 21]. Data shown as ± SEM. * P ≤ 0.05. Wilcoxon rank-sum test performed on non-infected vs low CAA, or non-infected vs high CAA, or low CAA vs high CAA for each time point separately D0, M7, or M12. Non-infected- light grey, low CAA—blue, and high CAA—dark grey. (C) Linear regressions fit between Hepatitis B titers and IgA+ ASCs, adjusted for sex, and student t-tests evaluated for the significance of the association. t (t-statistic). P ≤ 0.05 was considered significant. (Shape: triangle-Male, circle-Female; color: grey- non-infected, black- low CAA, blue- high CAA). (TIF) [file pntd.0011089.s004.tif]

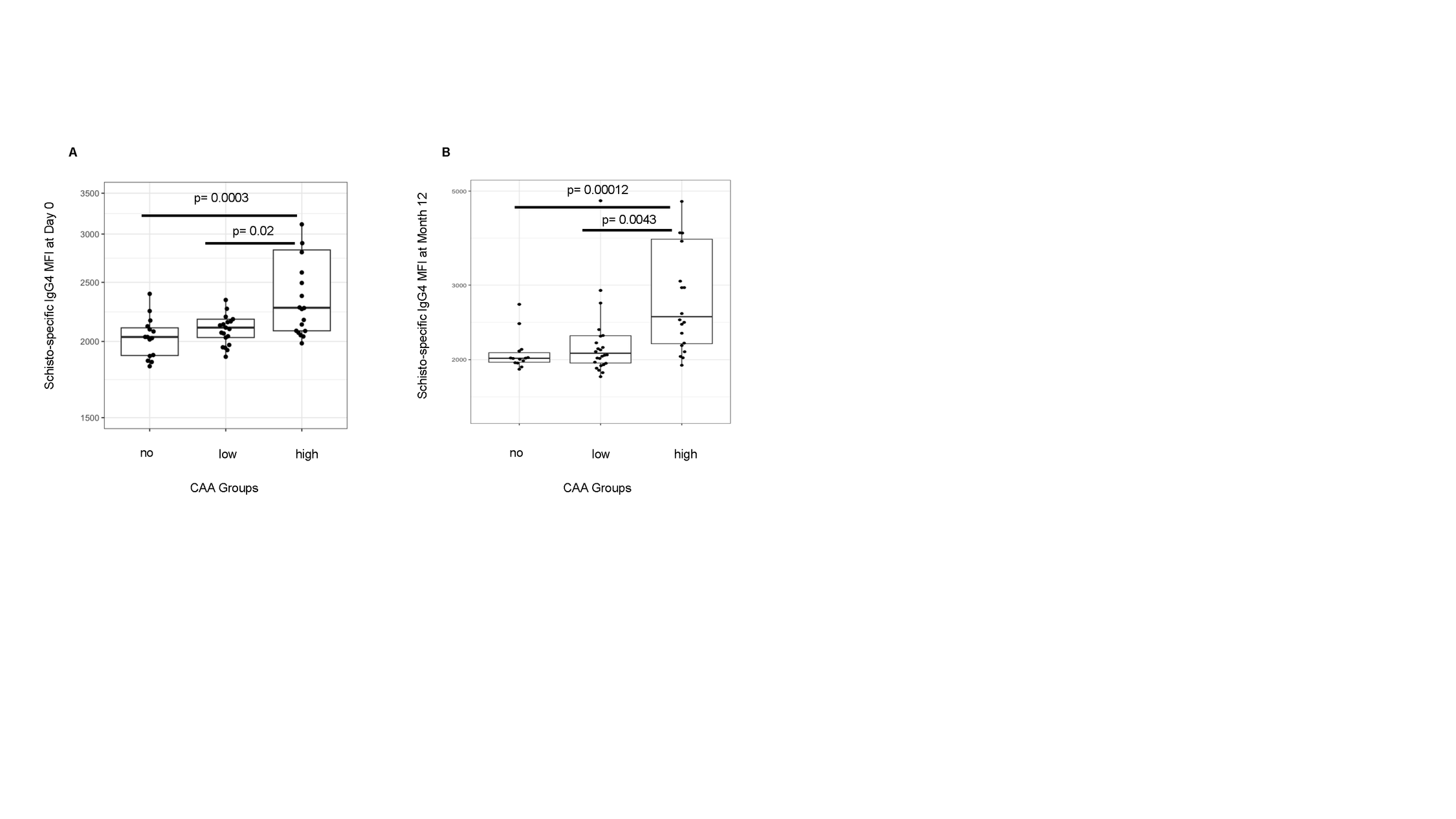

Supplement: S5 Fig — The mean Florescence intensity (MFI) of serum S. mansoni-specific IgG4 at (A) D0 [non-infected no, n = 15, low CAA, n = 21, and high CAA, n = 20], (B) M12 post-vaccination [non-infected no, n = 14, low CAA, n = 26, and high CAA, n = 20]. A student t-test was used to evaluate for the significance of the correlation. P ≤ 0.05 was considered significant. (TIF) [file pntd.0011089.s005.tif]

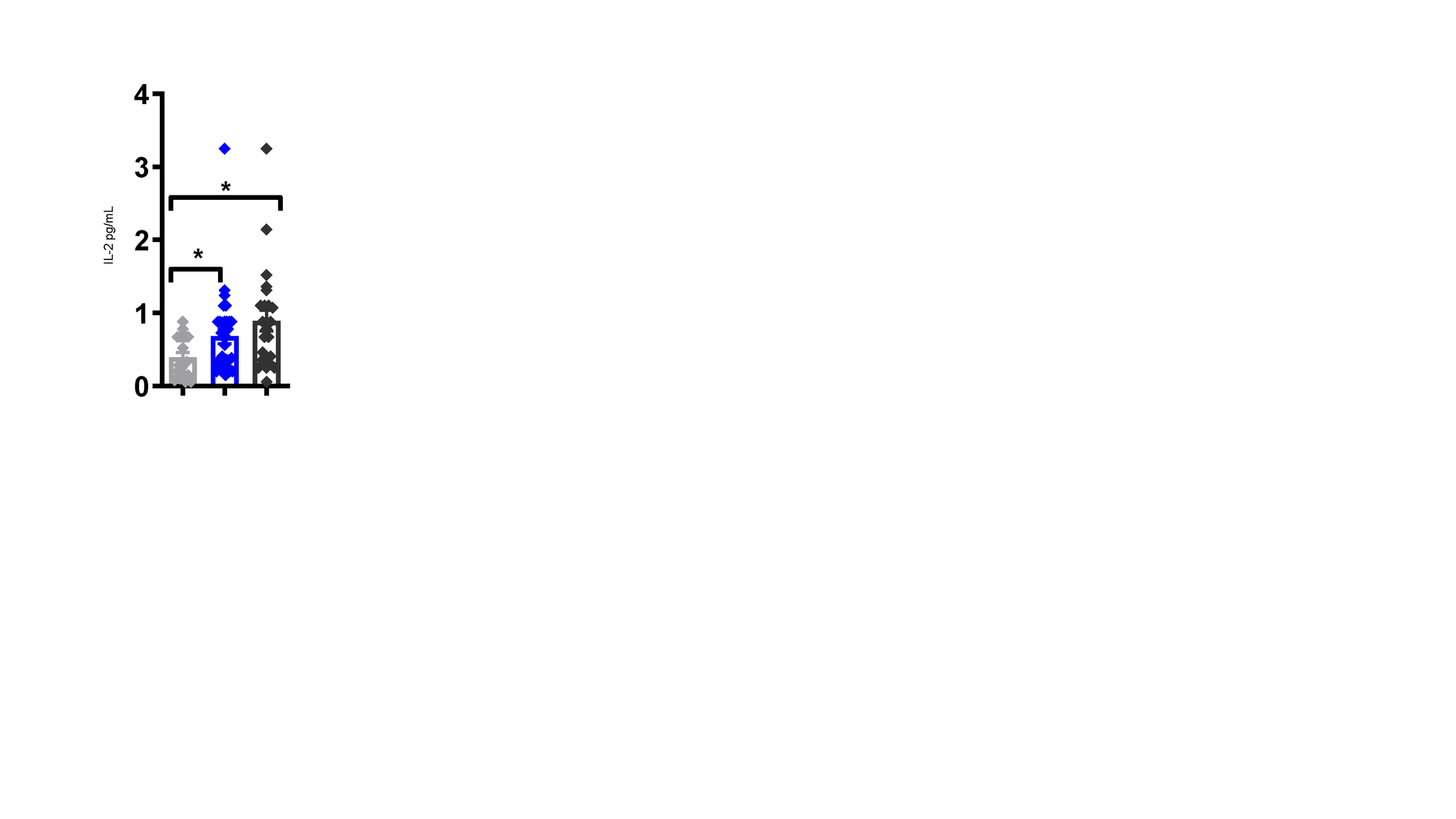

Supplement: S6 Fig — IL-2 levels in the plasma of non-infected, n = 19, low CAA, n = 32, and high CAA, n = 24 individuals pre-vaccination (day 0). Data shown as ± SEM. * P ≤ 0.05. Wilcoxon rank-sum test performed on non-infected vs low CAA, or non-infected vs high CAA, or low CAA vs high CAA for each time point separately. Non-infected- light grey, low CAA—blue, and high CAA—dark grey. (TIF) [file pntd.0011089.s006.tif]
